# Recommending reaction conditions with label ranking†

**DOI:** 10.1039/d4sc06728b

**Published:** 2025-02-03

**Authors:** Eunjae Shim, Ambuj Tewari, Tim Cernak, Paul M. Zimmerman

**Affiliations:** a Department of Chemistry, University of Michigan Ann Arbor MI USA paulzim@umich.edu; b Department of Statistics, University of Michigan Ann Arbor MI USA; c Department of Electrical Engineering and Computer Science, University of Michigan Ann Arbor MI USA; d Department of Medicinal Chemistry, University of Michigan Ann Arbor MI USA

## Abstract

Pinpointing effective reaction conditions can be challenging, even for reactions with significant precedent. Herein, models that rank reaction conditions are introduced as a conceptually new means for prioritizing experiments, distinct from the mainstream approach of yield regression. Specifically, label ranking, which operates using input features only from substrates, will be shown to better generalize to new substrates than prior models. Evaluation on practical reaction condition selection scenarios – choosing from either 4 or 18 conditions and datasets with or without missing reactions – demonstrates label ranking's utility. Ranking aggregation through Borda's method and relative simplicity are key features of label ranking to achieve consistent high performance.

## Abstract 2

Label ranking is introduced as a conceptually new means for prioritizing experiments. Their simplicity, ease of application, and the use of ranking aggregation facilitate their ability to make accurate predictions with small datasets.

## Graphical abstract

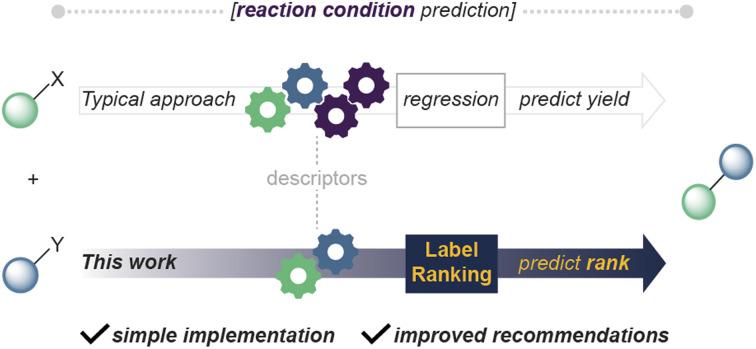

## Introduction

**Fig. 1:**
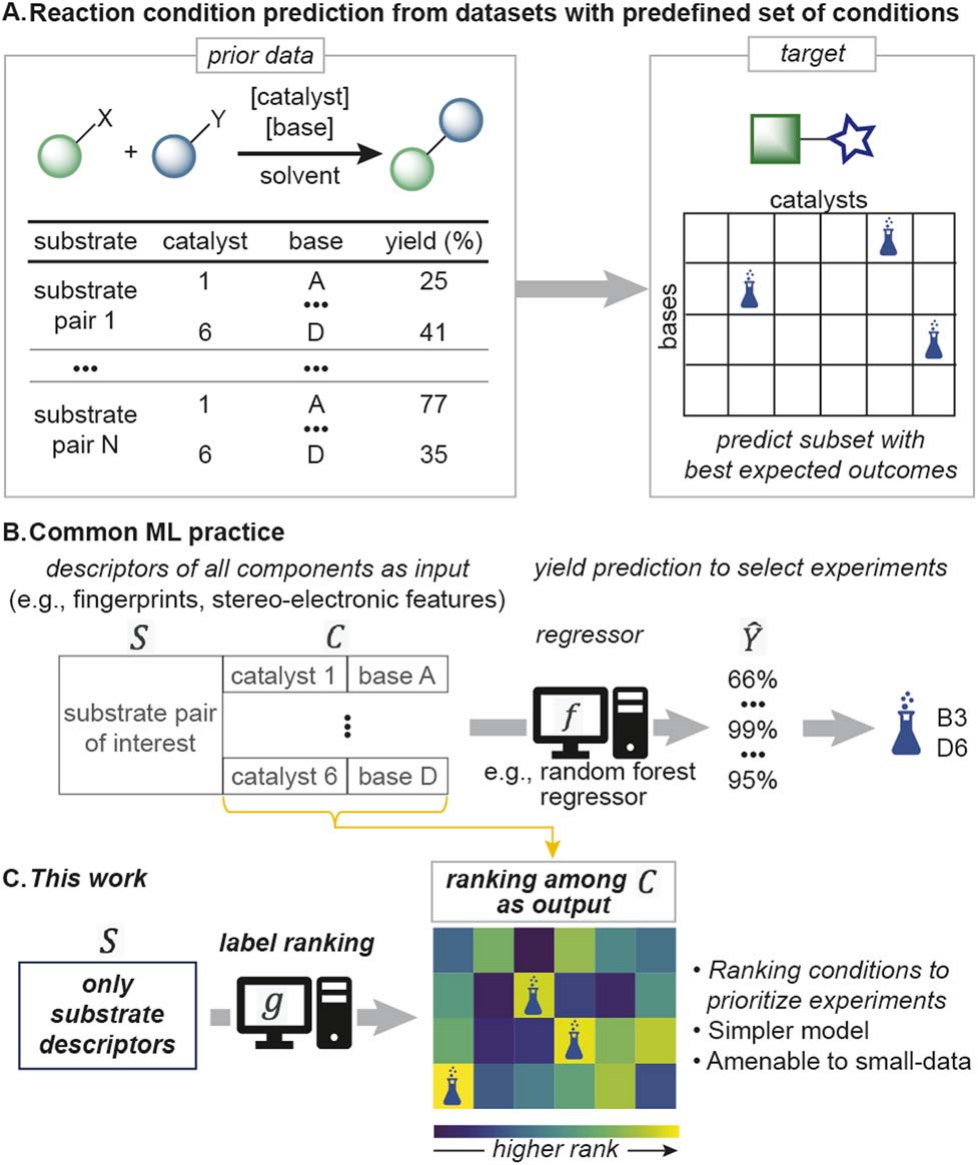
(A) Reaction condition prediction from a predefined set. (B) Currently, regressors are used to select reaction conditions. (C) This work approaches the problem differently, by ranking the reaction condition candidates using label ranking models.

Choosing reaction conditions is a routine yet important task for organic chemists.^1^ This task is non-trivial, especially for reactions that require different conditions for different substrates.^2–6^ The literature, user guides,^7,8^ or advice from experienced chemists can help shortlist conditions. Being able to narrow down the possibilities further to an even smaller number (*k*) of the most promising experiments would be practically useful (Fig. 1A).^7,8^ The key to successful reaction condition prediction therefore is to select the *k*-best conditions, which amounts to a ranking problem.^9,10^

Machine learning (ML) has demonstrated promise for decision making in organic synthesis.^11–16^ Most ML approaches to prioritizing reaction conditions^17^ have focused on quantifying yield or selectivity.^18–20^ For example, yield is modeled as a function of substrates and reagents using multivariate regression techniques: *Y* = *f*(*S*,*C*), where *Y* is predicted yield, and *S* and *C* denote substrate and reaction condition descriptors, respectively (Fig. 1B). While viable, this approach does not directly model the primary goal – how reaction conditions perform relatively to each other – and success highly depends on the regressor's precision. Furthermore, yield predictions involving unseen substrates can be unreliable, resulting in errors of >15% even with dense datasets.^21,22^ Alternative, simpler strategies could generalize better and improve ML's utility in the everyday problem of reaction condition selection.^23,24^

An intriguing alternative idea is to rank reaction conditions from a predefined list of conditions using only substrate features, *i.e.*, *C* = *g*(*S*) (Fig. 1C), which would reduce model complexity (*g*(*S*) is simpler than *f*(*S*,*C*)). Classification algorithms can in principle achieve this goal by treating the top-*k* reaction conditions for each training substrate as positive labels. However, in the typical scenario of sparsely labeled training datasets, substrates will have missing reaction conditions and the classifier may miss the top-*k* conditions. A classifier's practical utility will therefore be diminished in proportion to the number of missing datapoints. Alternatively to classifiers, label ranking (LR) is another strategy in the form *C* = *g*(*S*) that outputs rankings of candidate reaction conditions (see Label ranking algorithms)^9,25–28^ and is compatible with incomplete datasets. By reducing the intricacies involved with regressors and the demand for complete datasets compared to classifiers, LR could provide a practical tool for predicting reaction conditions with small datasets.

LR therefore is a novel strategy that could facilitate experimental campaigns by prioritizing effective reaction conditions without the need for extensive combinatorial datasets. Accordingly, we evaluate the utility of LR models against regressors and classifiers for selecting top reaction conditions from a larger, pre-selected list of possibilities. Relatively small datasets of synthetically important reactions are considered, including cases with missing reactions.

### Label ranking algorithms

LR refers to a class of ML algorithms that predicts rank across a predefined set of labels (reaction conditions) given features of an example (substrate). LR is distinct from the standard data science problems of classification and regression yet sits conceptually between the two. Binary classifiers predict from two choices – yes or no – while predictions from regressors can be any number. LR, on the other hand, needs to choose from all possible orderings amongst a finite set of labels. As this output space is discrete, it is simpler than regression. Simultaneously, with more possible outcomes than classification, LR can capture more nuanced relationships.^27^

**Fig. 2:**
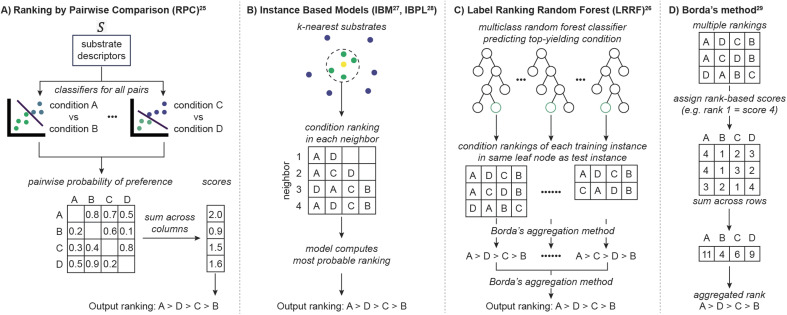
(A)–(C) Label ranking algorithms considered in this study. (D) Borda's method, a rank aggregation method used in LRRF.

One of the two main components of LR is learning from substrates using ML models. For example, ranking by pairwise comparison (RPC, Fig. 2A) learns to predict higher yielding conditions for a substrate across all possible pairs of conditions.^25^ RPC employs probability-based classifiers such as logistic regressors or random forests (in this work random forests are used, see Table S2†) to compare the pairs. Another ML technique involves instance-based probabilistic models (IBM^27^ or IBPL,^28^Fig. 2B), which are related to nearest-neighbor models. These identify substrates from the training data which are most similar to the query, assuming substrate feature similarity implies reactivity similarity. Alternatively, label ranking random forest (LRRF, Fig. 2C) utilizes random forest classifiers to predict the highest yielding condition.^26^ For any choice of LR algorithm, a new reactant enters the model and produces multiple pieces of information (pairwise preferences in reaction conditions, multiple neighbors, and training instances with the same best conditions for RPC, instance-based models and LRRF, respectively). In order to output a single prediction, these choices need to be combined into a single ranking, which is achieved in the second component of LR algorithms.

LR's second component aggregates the multiple pieces of information into a ranking of reaction conditions. Despite the task's simplicity, there is not a universal strategy for producing an optimal ranking. Among numerous approximations, Borda's method (Fig. 2D) has often been employed in LR because of its efficiency, availability of a modified version that deals with missing data,^29^ and competitive performance against other aggregation schemes.^30^ Borda's method, used in LRRF, assigns a score to every reaction condition proportional to its placement. Then, the final output ranking is determined by sorting the total score each condition collects across the multiple rankings. A variant of this process is used in RPC, where scores are assigned based on the probability of one condition to be preferred over another. IBM and IBPL utilize probabilistic models to compute the most likely ranking given the rankings of nearest neighbors. Therefore, LR is a modular framework where different models and aggregation strategies can be combined to predict rankings between reaction conditions.

LR algorithms are structured such that predictions involving all reaction conditions can be generated even if training data is missing for some substrates. This is because a model can fill in the gap by applying what has been learned from the labeled data (RPC, IBM and IBPL), or imputing a score that corresponds to the middle rank ((total number of conditions + 1)/2) in place of the empty entries during aggregation (LRRF). This suggests LR models can be trained in a data efficient manner,^31^ which would be practical for situations where all possible substrate–reaction condition pairs have not been evaluated. To evaluate LR's utility, various well-structured, synthetically-relevant reaction datasets were collected from the literature.

### Datasets

The reaction condition prediction problem appears in different chemical contexts. For well-known reactions, the list of conditions to choose from is relatively small. On the other hand, relatively complex reactions involve more choices and more experiments must be conducted to secure hits for new substrates. Being able to include the best reaction condition in these situations would facilitate experiment planning and improve reaction outcome. Accordingly, to evaluate viable algorithms in both situations, the following reaction datasets were selected. The datasets were originally curated for the purpose of either: (1) Developing reaction outcome prediction strategies, or (2) Maximizing the chance of obtaining high yields for a variety of substrates.

#### Datasets with a small set of reaction conditions

**Fig. 3:**
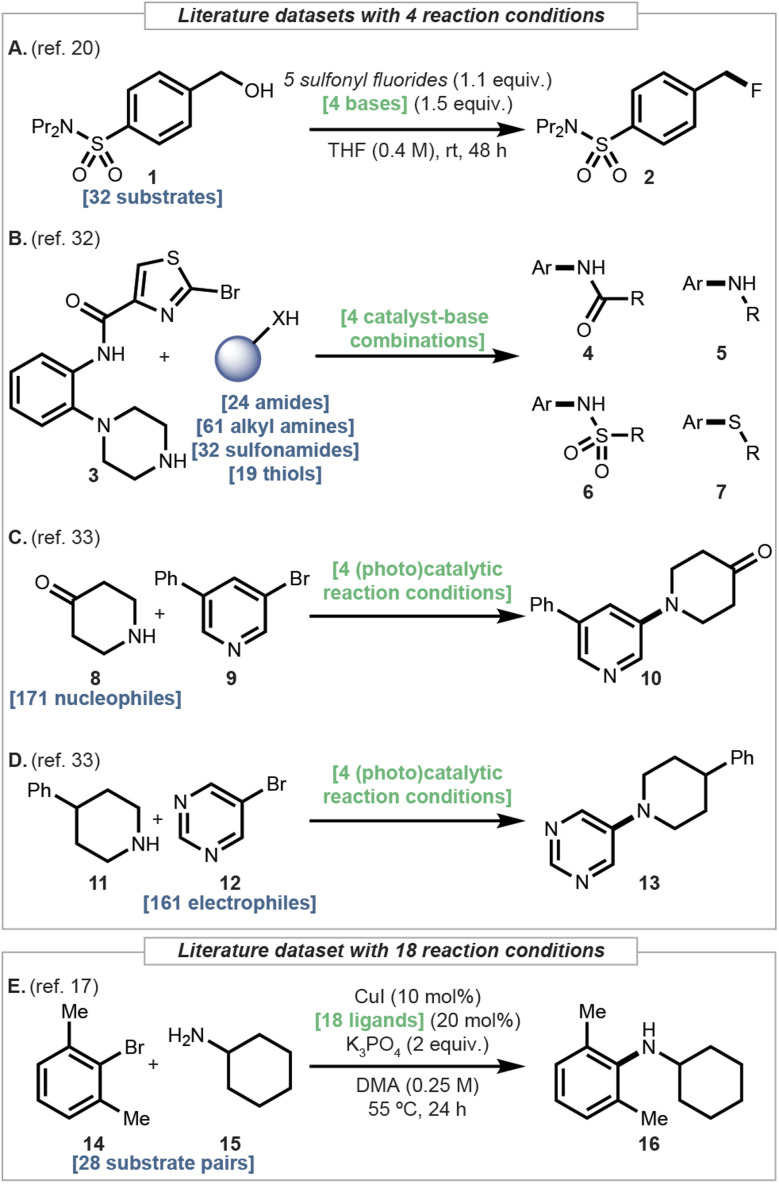
Datasets of this study. Bold blue text denotes the number of substrates. Bold green text shows the number of reaction condition candidates.

Introduction of a fluorine to a molecule can positively impact its physicochemical properties. As hydroxy groups are easily accessible, deoxyfluorination is a useful transformation. To evaluate the utility of regressors in yield prediction, a deoxyfluorination dataset comprising 32 structurally diverse alcohol substrates subjected to four bases and five sulfonyl fluorides was previously curated (Fig. 3A).^20^ Out of the 20 possible reagent combinations, choosing the most reactive sulfonyl fluoride with the four bases includes the top-1 reaction condition >50% of the time. To make this dataset a non-trivial example, five equally split subsets were considered, predicting the best base for a given sulfonyl fluoride.

C–heteroatom coupling reactions play an important role in synthesizing pharmaceutically relevant molecules. Despite the plethora of mechanistic studies and catalyst development for palladium-catalyzed reactions, determining an effective reaction condition is still considered difficult for new substrate pairs. One high-throughput experimentation (HTE) campaign screened four promising reaction conditions for various classes of nucleophiles to determine the highest yielding one (Fig. 3B).^32^ Specifically, three sets of nitrogen nucleophiles – 61 primary alkyl amines, 32 sulfonamides and 24 amides – along with a set of 19 thiol nucleophiles, each subjected to different sets of four reaction conditions, were considered.

In another study, C–N coupling reactions with four distinct catalytic systems (Cu, Pd, Ir/Ni- and Ru/Ni-photoredox) were compared.^33^ Two sets of >160 substrate pairs were subject to the four reaction conditions (Fig. 3C and D). Typical of HTE campaigns surveying numerous substrates, raw analytical measurements are reported because measuring calibration curves for each product is impractical.^30^

#### Datasets with more reaction conditions

The copper-catalyzed Ullmann C–N coupling reaction can be a useful alternative to the Pd-catalyzed counterpart. However, effective recipes have been reported on a case-by-case manner and the reaction mechanism remains elusive. It is therefore challenging to predict suitable reaction conditions for different substrates. As an effort to gain predictivity in this problem, a recent study curated a dataset of 28 substrate pairs subject to 18 ligands (Fig. 3E).^17^

Three other reaction datasets – nickel-photoredox catalyzed C–N coupling of complex aryl halides,^34^ iridium catalyzed C–H borylation^35^ and nickel-catalyzed borylation of aryl (pseudo)halides^36^ – with >10 reaction conditions were also initially considered. However, models failed to learn meaningful relationships between reactions and their outcomes as confirmed with adversarial controls (see ESI Section 2†). These datasets were therefore removed from subsequent analyses.

Datasets considered in this study cover synthetically important transformations and span a range of sizes, from a dozen to a few hundred. Datasets surveying different numbers of reaction conditions will put LR to the test under diverse chemical contexts. Moreover, variables in the reaction conditions vary from a single component (Fig. 3A: bases, Fig. 3E: ligands) to combinations of two (Fig. 3B: catalyst and base) and even arbitrary combinations (Fig. 3C and D).

### Metrics

There is no unique way to access the quality of predicted ranks between reaction conditions. For example, top-1 accuracy measures how well the highest yielding condition is ranked as best by the model. It is also important, however, to understand the quality of choices when the best case does not make it in the top-*k* suggestions. In this context, mean reciprocal rank (MRR) complements the top-1 accuracy by calculating the reciprocal of the ground-truth rank, including all sub-optimal cases (*e.g.*, if the top prediction from RPC is actually the second best, its score will be 0.5). To further promote the practicality of MRR, reactions that failed to give product were further penalized to a score of 0.04 since obtaining sub-optimal yields is preferred to a yield of 0%. With MRR, random selection from four possibilities corresponds to a score of 0.52, which is the average of 1, ½, 1/3 and ¼. A perfect prediction results in a top score of 1.00 while the worst possible score is 0.25. Finally, the Kendall-tau coefficient provides a holistic assessment of the full ranking between all reaction condition candidates. Predictions that perfectly rank all reaction conditions give a value of 1 while random guesses correspond to 0 and the lowest possible score is −1. As MRR best describes the practical utility of a model's top suggestions, MRR is mainly used to discuss model performances. Top-1 accuracy scores and Kendall-tau coefficients are presented in the ESI.† All reported scores are averages across cross-validation (CV) folds (see Sections S1.2.1 or S2.2† for details).

### Predicting the highest yielding reaction condition from four candidates

#### Using fully combinatorial datasets

The initial investigation focused on comparing the utility of different algorithms for choosing the top-yielding condition from four candidates (datasets in Fig. 3A–D). For models based on random forests (regressor, classifier, LRRF and RPC), reactions were represented with physical descriptors (see ESI Page S4†). In contrast, because nearest neighbors computed from raw physical descriptors may not be as meaningful for deducing reactivity similarity, count-based Morgan fingerprints of 1024 bits and radius of 3 were used for instance-based models (KNN, IBM and IBPL; see Fig. S7 and Table S3† for adversarial controls^37,38^). The final recommended reaction condition was selected as follows. For each test substrate, RFR predicted yields for every reaction condition candidate and the one with the highest predicted value was used. On the other hand, for RFC, a multi-class classification problem was formulated. A multi-class RFC gives a single prediction out of 1–4, which condition will give highest yield, given a substrate. The predicted class for each substrate was selected. For LR, the condition at the highest rank was chosen. The baseline corresponds to singly selecting the reaction condition that gives the highest average yield across the training data, which is expected to fall short for transformations that require different conditions for different substrates.

**Fig. 4:**
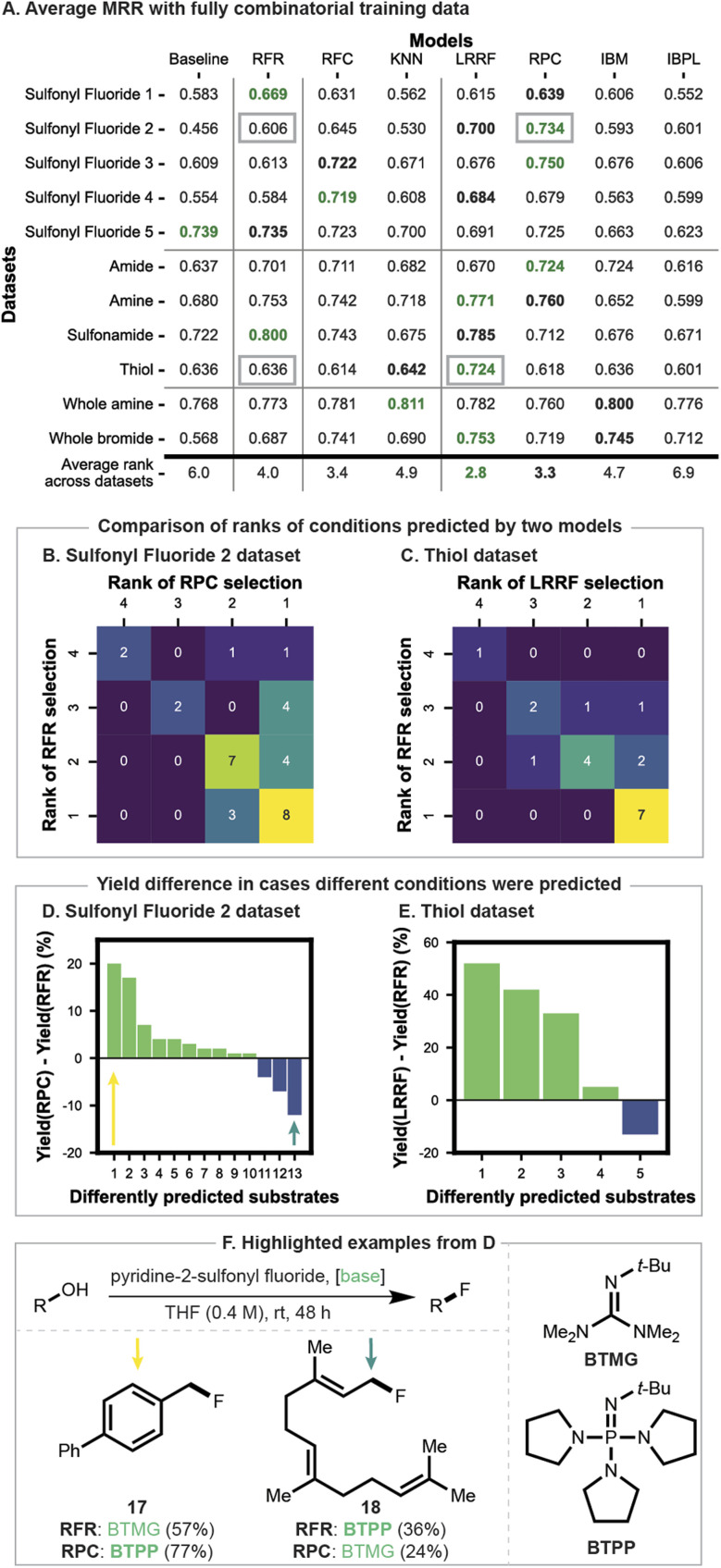
(A) Model performance evaluated by average MRR across CV folds. Vertical lines classify algorithm types (baseline, regressor, classifiers and LR, from left to right). Green and bold black numbers correspond to the top and second-best performants in each dataset, respectively. (B and C) Number of pairs of ranks of conditions predicted by two models with grey squares in (A) rows 2 and 9, respectively. (D and E) Resulting yield difference for cases where different conditions were predicted. Green and blue bars correspond to substrates where RPC or LRRF and RFR made better choices, respectively. (F) Examples of different predictions from (D).

The mean reciprocal rank (MRR) achieved by each model with each dataset is shown in Fig. 4A (top-1 accuracy scores and Kendall-tau coefficients can be found in Fig. S5 and S6†). The first five rows correspond to the deoxyfluorination dataset depicted in Fig. 3A. The baseline struggles to make meaningful suggestions since the best performing bases vary by substrate. The random forest regressor (RFR) outperforms the baseline in four cases, and the random forest classifier (RFC) outperforms RFR in three cases (rows 2–4). Two LR algorithms based on random forests – LRRF and RPC – outperform RFR in three cases (rows 2–4). RPC, in particular, achieves higher MRR than RFC in four datasets (rows 1–3 and 5). Instance-based models show relatively lower performances, rarely outperforming RFR. These results suggest that while around 100 fully combinatorial training datapoints may be enough to train effective regressors, both classifiers and LR can be useful alternatives for choosing good reaction conditions.

The next four rows of Fig. 4A show model performances on the C–heteroatom coupling reactions with four nucleophiles (Fig. 3B). As small datasets with different conditions being preferred for different substrates, similar trends are observed with the first five rows. The baseline shows mediocre performance compared to other strategies. RFR returned mixed results, coming at 3^rd^ or 4^th^ place for nucleophiles other than sulfonamide, where it came on top. RFC returned higher MRR than RFR only for amides with a relatively small improvement. LRRF and RPC performed well overall, being within the top-2 for three and two datasets, respectively. Instance-based models, on the other hand, showed generally poor performance, although IBM scored decently for the amide dataset. These results imply effective reaction conditions can be selected in the low-data regime (as few as 19 substrates), with LR models showing high placements.

The last two rows of Fig. 4A assess models on datasets in Fig. 3C and D. These datasets are distinctly larger than the previous datasets, up to nine times the size (171 substrates). For the amine dataset, the baseline performs well. All RF-based models struggled to score a meaningfully higher score, being outperformed by KNN and IBM. In contrast, the baseline selection criteria performed the worst on the bromide dataset. While RFR outperformed it, it only matched KNN in terms of performance. LRRF overall made best recommendations with IBM, RFC, and RPC with slightly lower scores. In all, while instance-based models seem to perform well with larger datasets, differences in performance across all models were relatively small.

Across all datasets evaluated, there was no consistently superior algorithm. While RFR returned a higher average rank compared to all instance-based models, it was outperformed by alternative models. LRRF was the overall top performant, followed by RPC and RFC, supporting them as useful strategies for selecting the best condition from four candidates when a fully combinatorial dataset is in hand. Although these conclusions from evaluating model performance with MRR are useful, analyses in the subsequent two paragraphs show how these scores translate to an experimental campaign.

Fig. 4B and C compare two pairs of models (RFR *vs.* RPC and LRRF, respectively), both differing in MRR by 0.1. To understand what this means for predicting the highest-yielding conditions, the quality of recommendations was compared. Along the diagonals are the number of substrates where the two models predicted the same reaction conditions. Off-diagonals correspond to cases where RFR predicted better conditions than the other model (below diagonal) or worse (above diagonal). In both datasets, conditions recommended by RFR and the other model were simultaneously among the better half (bottom right quadrants) for ∼65% of substrates. The alternate model, however, suggested better reaction conditions than RFR more frequently (10 *vs.* 3 and 4 *vs.* 1 in Fig. 4B and C, respectively). Among them are cases where RFR predicted one of the two lower yielding reaction conditions, while RPC or LRRF identified the best (five substrates in Fig. 4B and one in Fig. 4C). Predictions of lower rank would result in lower yields, so this aspect was quantified next.

Model-specific differences in yield for individual substrates are shown in Fig. 4D and E. The blue and green bars correspond to the yield benefit and detriment of using RFR over the other model. These comparisons reveal specific substrate(s) with RPC and LRRF achieving nearly 20% and 50% higher yield over RFR, respectively (leftmost green bar). For the remaining substrates in Fig. 4D, the benefit is less than 10%. When RFR suggested a better condition, a similar pattern was observed with the highest benefit (rightmost blue bar) being smaller. Although detailed distributions differ by datasets, these observations generally hold for comparisons between RF-based models (Fig. S7–S17†). As such, in cases where cumulative benefit across multiple substrates is important, higher-performing LR algorithms should be prioritized.

Lastly, specific predictions with largest yield differences in Fig. 4D are shown in Fig. 4F. When pyridine-2-sulfonyl fluoride is the fluorination reagent, RPC and RFR suggested one of the bulkier bases, presumably recognizing the major structural aspect (sterical accessibility) of the substrates. The models did not, however, uniformly capture the more subtle feature (benzylic 17*vs.* allylic 18).

#### Using incomplete datasets

Although possession of fully combinatorial datasets is advantageous in model building, sparsely populated datasets are more common. Literature reports are representative sources of such data where arbitrarily selected substrates are evaluated under different reaction conditions. Under such sparsity – substrates being tested with as low as two reaction conditions – learning relative preferences would be more practical than the alternatives: predicting specific yield values, or classification which requires knowing the positive label. Therefore, the utility of LR against typical classifiers and regressors under this situation was evaluated next. To simulate missing data, results for two out of four reaction conditions for every reactant were randomly masked in the training data (see Section S1.3† for results when one reaction is masked). For each CV fold, 10 different sets of data removals were used. Average MRR values across all evaluations were measured (see Fig. S22 and S23† for top-1 accuracy scores and Kendall-tau coefficients).

**Fig. 5:**
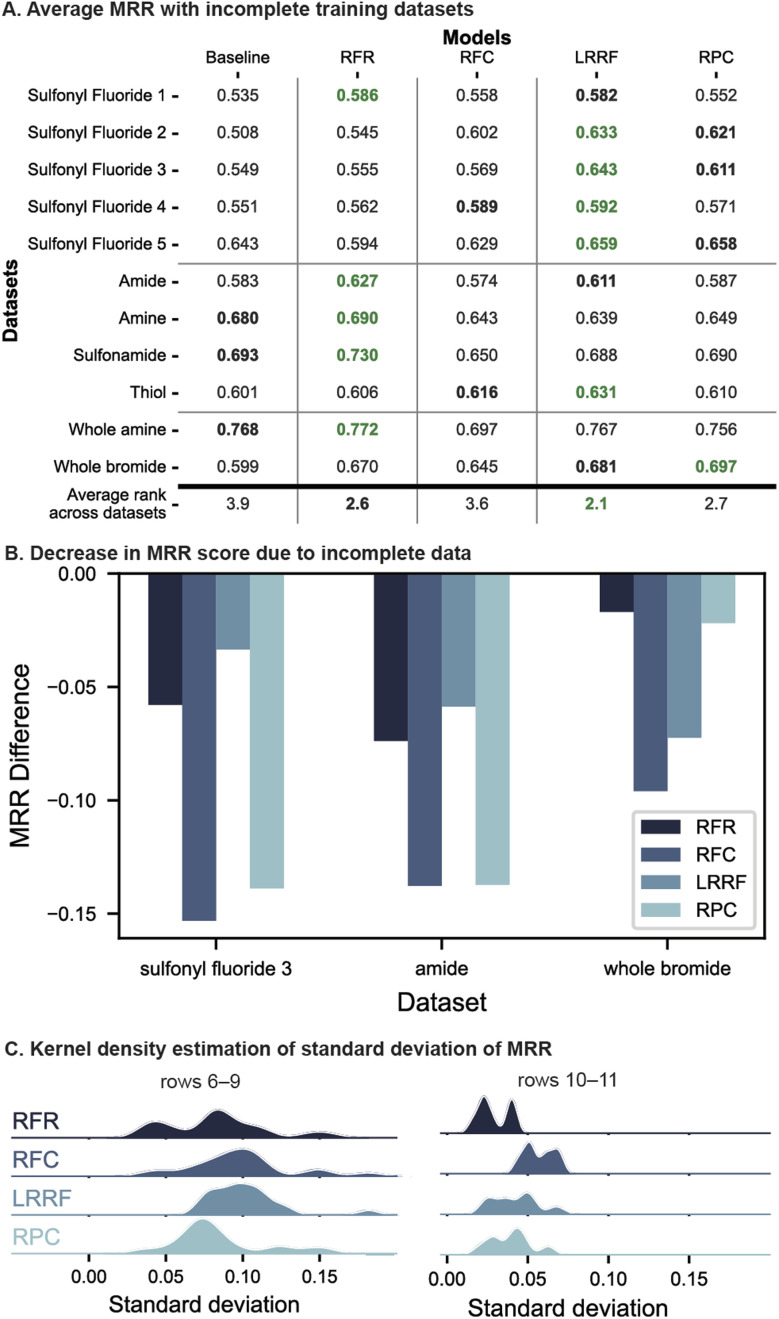
(A) Model performance on datasets where each substrate is missing 50% of reactions. Green and bold black numbers correspond to the top and second-best performants in each dataset, respectively. (B) Comparison of MRR degradation between models. Three datasets with the largest detriment are shown. (C) Kernel density estimation plots of standard deviation of MRR across data masks, collected across CV folds and datasets.

Overall, LRRF returned the highest average rank across all datasets, followed by RFR and RPC, which differed by small amounts. LR algorithms particularly performed well on the first and last sets of evaluations (Fig. 5A rows 1–5 and 10–11), underperforming RFR in only two cases (rows 1 and 10). Other than these two, LRRF was either the top (rows 2–5) or the second-best performant (rows 1 and 11). RPC outperformed LRRF in one case (row 11), although the difference in average MRR was small (<0.016). Thus, LR remains an effective strategy to select effective reaction conditions under low-data situations.

RFR shows performance comparable to LR with a high average rank of 2.6 and being the top performant in five cases (Fig. 5A). A significant portion of this overall rank stems from the high placement with all of C–heteroatom coupling datasets (rows 6–9) where an MRR difference of up to 0.051 is observed compared to LR models. This consistency may be attributed to these datasets' structure of the four reaction conditions which are combinations of two catalysts and two bases. RFR can leverage this toward predicting yields since reagent information is included in the input unlike LR where the reagents are treated as outputs that cannot share information with each other. As such, RFR may be the algorithm to use over LR when the reaction condition candidates are combinatorial in reagents, particularly when a large portion of data is missing (when only one reaction condition was masked, in two out of the four datasets, LRRF showed higher MRR scores than RFR although differences were small. See Fig. S19†).

Data sparsity presented a challenge for all models and thus lower MRR than Fig. 4A are observed, up to a decrease of 0.15 MRR score (Fig. 5B). Most notable is the degradation of RFC's performance from being comparable to LR with full datasets to only slightly better than the baseline in terms of average rank across incomplete datasets (3.6 *vs.* 3.9). This failure of RFC with incomplete datasets is likely due to the ground-truth best condition being part of the masked data. This causes the positive labels to be marked with suboptimal conditions, thwarting RFC's learning of the best one. Regressors, in contrast, does not suffer from this problem as they are trained on yields of each reaction, and thus are one of the two less-degraded models in Fig. 5B (this observation holds in 8 out of 11 datasets, see Fig. S24†). Relative outcomes, which RPC learns from (Fig. 2A), are ideally also not impacted by missing datapoints. However, with 50% of the data missing, the number of examples to learn pairwise preferences from drops threefold (6 *vs.* 2) and can result in a relatively high performance degradation (Fig. 5B, left two columns). In comparison, although LRRF is dependent on the ground-truth best condition due to the base model being RFC, the impact on performance is mitigated (Fig. 5B) by the ranking aggregations (LRRF's MRR degradation is the lowest in 4 out of 11 datasets, see Fig. S24†).

The impact of missing data was further investigated by a kernel estimate of the MRR distribution across the 10 dataset masks (Fig. 5C, *c.f.* Fig. S26†). In both plots, RFC's distribution of MRR values is larger than other algorithms, which is expected from models trained on suboptimal labels. LRRF's distributions, while also larger overall than RFR and RPC, is notably on the lower side compared to RFC, supporting Borda's aggregations mitigating prediction variance.

Altogether, LRRF presents effective ways for selecting high-yielding reaction conditions from four choices regardless of with and without missing data. This is in contrast to conventional RFR and RFC, which fell relatively short under particular scenarios – when the available datasets were fully combinatorial and incomplete, respectively.

### Predicting conditions from a larger number of possibilities

Dealing with relatively less studied transformations involves conducting multiple reactions to search for the right conditions. While this goal is shared with prior examples over a focused set of possibilities, the larger number of reaction conditions at play presents a challenge for regressors and ranking algorithms alike. Accordingly, the study was extended to evaluate algorithms on the Ullmann dataset with 18 ligands (Fig. 3E; results using other datasets and adversarial control studies are presented in Fig. S32–S36†). To simulate a practical reaction condition search situation, we assumed about 25% of the candidates can be tested. Accordingly, each model was allowed to select four ligands for evaluation. While label ranking algorithms and RFR were trained in the same manner as the previous section, a multi-label RFC was used for selecting multiple reaction conditions. In contrast to multi-class RFC that learns to map a substrate to its best reaction condition, multi-label RFC aims to connect the substrate to the top-*k* reaction conditions simultaneously. To ensure four reaction conditions are recommended, the top-4 reaction conditions with the highest probability of returning positive labels were selected. The top-1 accuracy and reciprocal ground-truth rank of the highest yielding ligand selected by each model were computed and averaged across CV folds (see Section S2.2† for details).

**Fig. 6:**
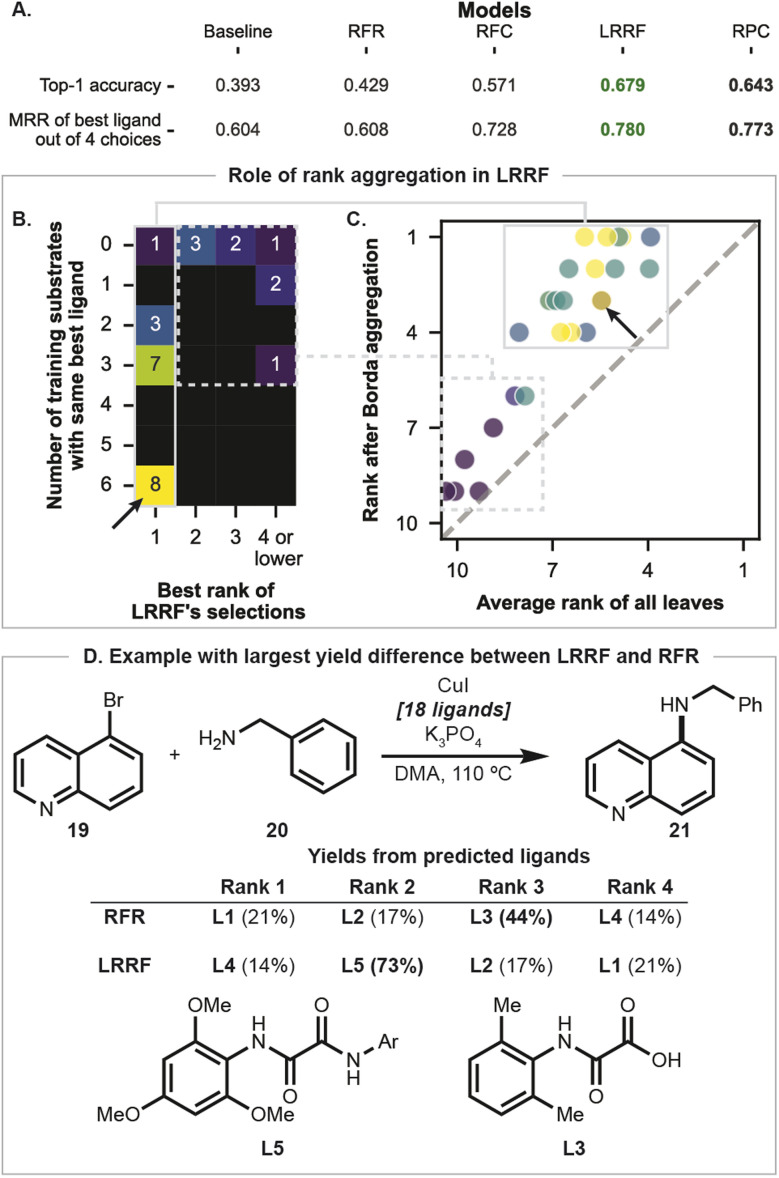
(A) Performance of each algorithm on the Ullmann dataset evaluated by top-1 accuracy and MRR across CV folds. Green and bold black numbers correspond to the top and second-best performants in each dataset, respectively. (B) Dependence of the quality of LRRF's prediction for each substrate pair (columns) on the number of training substrates that share the best ligand (rows). (C) Predicted ranks of the best ligand for each test substrate averaged across leaf nodes in LRRF's base RFC are compared against ranks after the first Borda aggregation. (D) Example with the largest yield difference between ligands predicted by RFR and LRRF.

For this challenging dataset with different ligands returning highest yields for different substrates, RFR was only comparable to the baseline (Fig. 6A; top-1 accuracy difference of 0.036 corresponds to one substrate out of 28; see Fig. S37 and S38† for further analysis). In contrast, RF-based LR algorithms performed well when searching amidst 18 possible ligands. Particularly, LRRF's top-1 accuracy and MRR scores were higher than RFR's by 0.150 and 0.172, respectively (Fig. 6A). Notably, there were seven substrates for which LRRF identified the best ligand while RFR could not, but none the other way round (Fig. S39A†).

To provide insight into LRRF's performance, LRRF predictions across all CV splits were interrogated. LRRF's predictions include the highest yielding ligand when two or more examples in the training set shared the same optimal ligand (Fig. 6C, rows marked 2 and below; 18 of 19 examples). One might imagine that LRRF's base RFC accurately identified these training substrates and this was the origin of the good performance. This is not the case, however, as the average of predictions from each decision tree situate the best ligand at the fourth place or lower (Fig. 6D, *x*-axis). Borda's aggregation improves the placement of the desired ligand (Fig. 6D, markers all above grey diagonal line), including a ligand with an average rank as low as 8 as one of the final four recommendations (blue marker at the left bottom of solid box in Fig. 6D). Combined, this suggests the importance of Borda aggregation in LRRF, reinforcing the base classifier which may be insufficient on its own.

Specific examples were interrogated to gain further insight into the differences between models. The reaction leading to 21 (Fig. 6D) showed the largest yield difference between the predicted best ligands (see Fig. S39A† for all yield differences when best predicted ligands differed between LRRF and RFR, and Fig. S39B† for the specific example where RFR's benefit is highest, by 7%). LRRF's prediction for coupling 19 and 20 included L5, the ground-truth best ligand, while RFR's best performing ligand was L3, which is actually 4^th^ best. This difference occurs while L1, L2 and L4 overlap between the two models' predictions. In fact, all predictions between RFR and LRRF have at least two ligands overlap (Fig. S40†), yet this still leads to a significant difference in both top-1 accuracy and MRR.

The more important consideration that needs to be made is whether a sufficient number of substrates have been studied compared to the number of reaction condition candidates. This is because LR models require sufficient data to learn relationships between substrates and relative reaction condition performance. Here, while LR was shown useful on the Ullmann dataset (which had 1.5× the number of reaction conditions of substrates) it failed to meaningfully surpass adversarial controls on aryl halide borylation datasets with a comparable substrate-to-reaction condition ratio (Fig. S35 and 36†).

## Discussion

Selecting the best reaction condition out of multiple candidates is a problem chemists face every day. Although development of generally applicable reaction conditions will mitigate this issue, it accompanies significant experimental exploration.^12,39–41^ When a dataset is already available, it is practical to leverage it to downsize experiments for a specific substrate. The standard practice is to choose conditions that give the best average results—*i.e.*, those that work well for a variety of substrates—but many cases remain where this choice becomes limiting. When a fully combinatorial dataset is in hand, a simple diagnosis can be done by counting the frequency of each condition being the top performant. If the most frequent condition accounts for less than half of the substrates in hand (which roughly corresponds to MRR ≦ 0.6), ML could be worthwhile to consider.

Currently, the most straightforward way to go about tailoring reaction conditions for new substrates is with regressors, by predicting yields from each condition candidate. RFR is widely used for this purpose, particularly on small datasets.^13,20,42^ For the task of prioritizing the reactions to try for new substrates, however, RFR was generally not effective. Even though predictions were all made on reaction conditions that RFR has been exposed to, precise differences between them were not accurately modeled in small datasets with a few dozen substrates.

Reformulating the problem to predicting the top-*k* conditions appears to us a more focused task than regression, and likely to be more successful. Not only are reagent descriptors no longer necessary, but also the goal becomes more tied to the practical question of ‘what is the best condition for this substrate?’. Among conventional ML models, classifiers like RFC have some efficacy, but herein RFC was only effective for the easiest scenario involving fully combinatorial datasets. The lack of the ability to cope with missing data and applicability to situations with more choices leaves room for alternative ranking strategies.

RF-based LR models consistently performed well in recommending the highest yielding reaction condition across different situations and datasets. LR was able to compete or outperform RFR by learning how conditions compare for different substrates. The aggregation of these multiple comparisons empowers LRRF and RPC to cope with missing data and prioritize useful conditions from a larger pool, even when conventional models fall short. For sensitive transformations like those studied here, high reactivity can only be achieved with certain conditions compatible with the substrate. In other words, substrates that have common high yielding conditions likely share key features that affect reactivity. Among LRRF and RPC, the assumption LRRF was developed upon – substrates that share the best condition are likely to be similar in the overall rank of conditions^26^ – makes chemical sense for conditions that give good yield. As such, LRRF is well-posed for reaction condition recommendation.

## Conclusion

By learning relative performances between reaction conditions, LR is a ML framework that is particularly well suited for predicting useful conditions for new substrates. Largely, LR was able to prioritize higher yielding reaction conditions better than RFR under a variety of situations. Particularly effective and consistent was LRRF, a LR variant of RFC that outputs ranking between reaction conditions from training substrates predicted to share the best condition. Key features supporting LRRF's performance were ranking aggregation (Borda's method) and its low dimensionality, due to reaction conditions being the output of the model, rather than part of its input. In all, the results of this work suggest that LR should be more widely considered for making decisions in organic synthesis.

## Data availability

Datasets used in this study were used as provided from multiple publicly available sources. These files, along with the code for reproducing this study can be found at https://github.com/ZimmermanGroup/LabelRanking. Data that support results discussed in this work can be found in the ESI associated with this article.†

## Author contributions

E. S., A. T., T. C. and P. M. Z. conceived the project. E. S. designed and conducted computational studies. A. T., T. C. and P. M. Z. supervised the work. E. S. and P. M. Z. wrote the paper with contributions from all authors.

## Conflicts of interest

The authors declare no competing financial interest.

## Supplementary Material

SC-OLF-D4SC06728B-s001
