# Supplementary material for: Recommending reaction conditions with label ranking
Source: Chem Sci. 2025 Feb 3;16(9):4109–18. doi: 10.1039/d4sc06728b (PMC11788591; doi:10.1039/d4sc06728b)
Supplement: SC-016-D4SC06728B-s001 [file SC-016-D4SC06728B-s001.pdf]

Supplementary Information (SI) for Chemical Science.
This journal is © The Royal Society of Chemistry 2025

Supporting Information for

Recommending reaction conditions with label ranking

Eunjae Shim[1], Ambuj Tewari[2,3], Tim Cernak[1,4], Paul M. Zimmerman[1,*]

1. Department of Chemistry, University of Michigan, Ann Arbor, MI
2. Department of Statistics, University of Michigan, Ann Arbor, MI
3. Department of Electrical Engineering and Computer Science, University of Michigan, Ann Arbor, MI
4. Department of Medicinal Chemistry, University of Michigan, Ann Arbor, MI

*paulzim@umich.edu

Code to reproduce all content of this work can be found at :
github.com/ZimmermanGroup/LabelRanking

Table of Contents

1) Datasets with four reaction conditions S-01
- 1-1) Dataset preprocessing, featurization, and distribution of best conditions S-01
- 1-2) Additional studies with fully combinatorial datasets with four reaction conditions S-06
- 1-3) When one of the four reaction conditions are missing S-23
- 1-4) Additional studies when two of the four reaction conditions are missing S-26

2) Datasets with a larger number of possibilities S-27
- 2-1) Dataset preprocessing, featurization, and distribution of best conditions S-30
- 2-2) Evaluation setup S-34
- 2-3) Results S-35
- 2-4) Adversarial controls S-36
- 2-5) RFR’s individual predictions on all CV folds of the Ullmann dataset S-39
- 2-6) Comparison of RFR’s and LRRF’s predictions on the Ullmann dataset S-40

3) References S-43

1. Datasets with four reaction conditions

1.1 Dataset preprocessing, featurization, and distribution of best conditions

1.1.1 Deoxyfluorination dataset (Figure 3A)

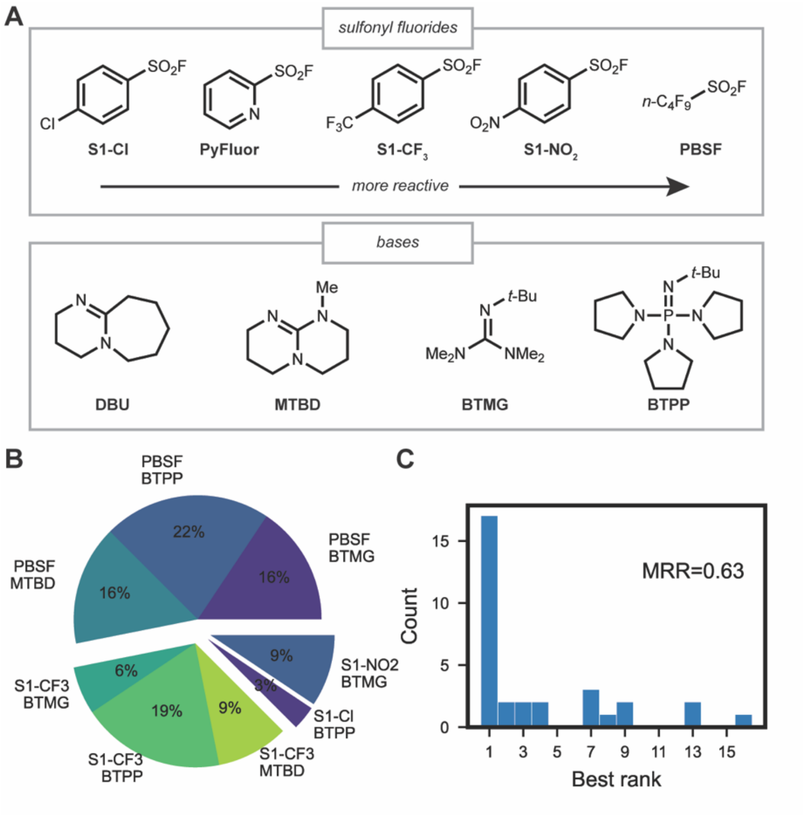

Figure S1. (A) Reagents used in the deoxyfluorination dataset. (B) Portion of substrates each reagent pair is best for. Each exploded portion corresponds to a sulfonyl fluoride. (C) Histogram of best rank when four bases are selected with PBSF.

The deoxyfluorination dataset[1] includes reactions of 32 alcohol substrates subject to a total of 20 reaction conditions, combining five sulfonyl fluorides and four bases (Figure S1A). Physical descriptors were used as features of all compounds as provided in the previous work.[1] Choosing the most reactive sulfonyl fluoride PBSF is an effective choice, being the best fluorination reagent for 17 out of 32 alcohols (54%, Figure S1B). Of the remaining 15 substrates where the best condition does not use PBSF, the best rank achieved among the four bases in combination with PBSF is still within the top-4 in six substrates (Figure S1C). Therefore predicting the best base for each sulfonyl fluoride was considered as a more difficult problem, and the dataset was divided up accordingly for subsequent analysis.

Figure S2 shows the portion of each base being the highest yielding with sulfonyl fluoride separation. While BTPP and MTBD are effective across all sulfonyl fluorides, there are at least five substrates where BTMG performs best, making the reaction condition prediction problem sufficiently interesting for tests of ML models.

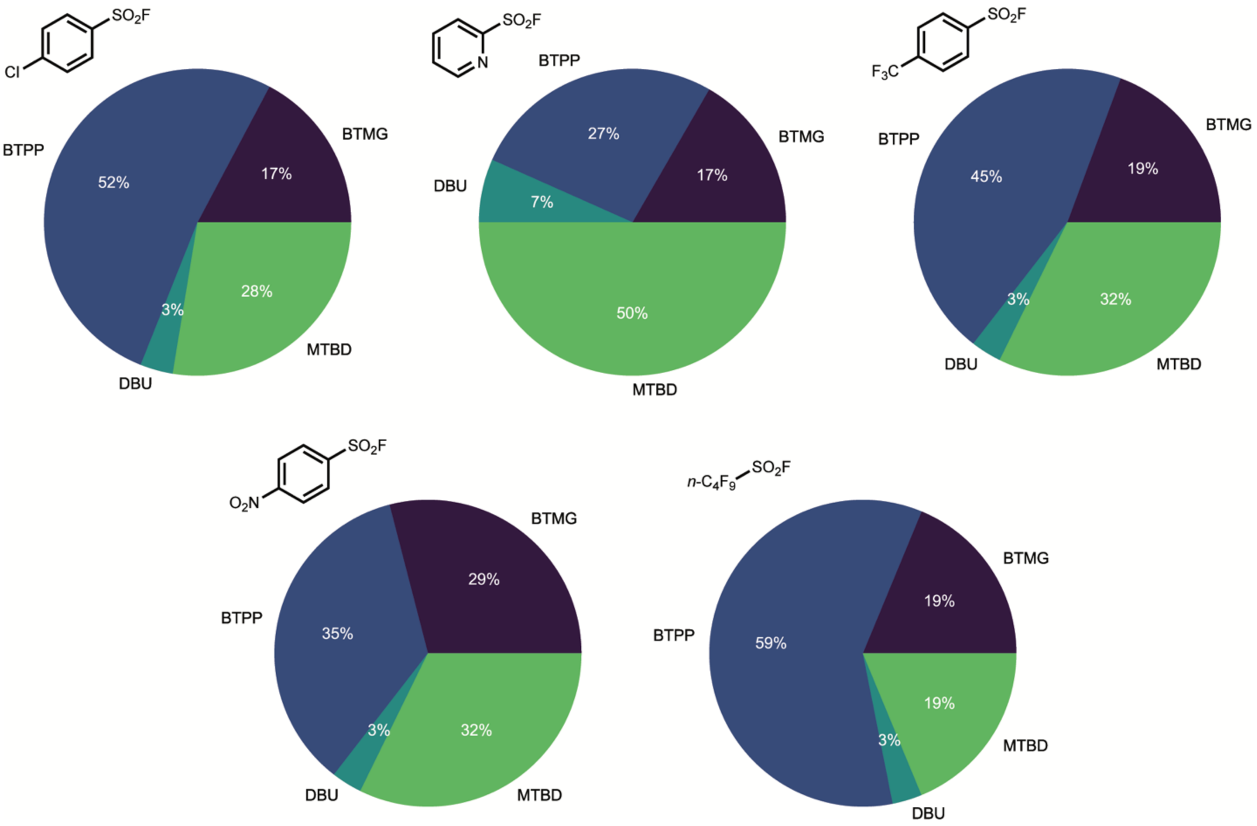

Figure S2. Portion of substrates in the deoxyfluorination dataset each base is the best for, for each sulfonyl fluoride.

1.1.2 C–X coupling dataset (Figure 3B)

This dataset explored the coupling of a pharmaceutically relevant molecule 2-bromo-*N*-(2-(piperazin-1-yl)phenyl)thiazole-4-carboxamide (**3**) with various types of nucleophiles.[2] A large number of reaction conditions (24 or 32 depending on nucleophile type) were evaluated on a handful of exemplary building blocks to narrow down to a focused set of four reaction conditions tailored for each nucleophile type. The latter portion was used in this study after preprocessing as described below. The distribution of

the top-performing conditions of each nucleophile dataset after preprocessing is shown in Figure S3.

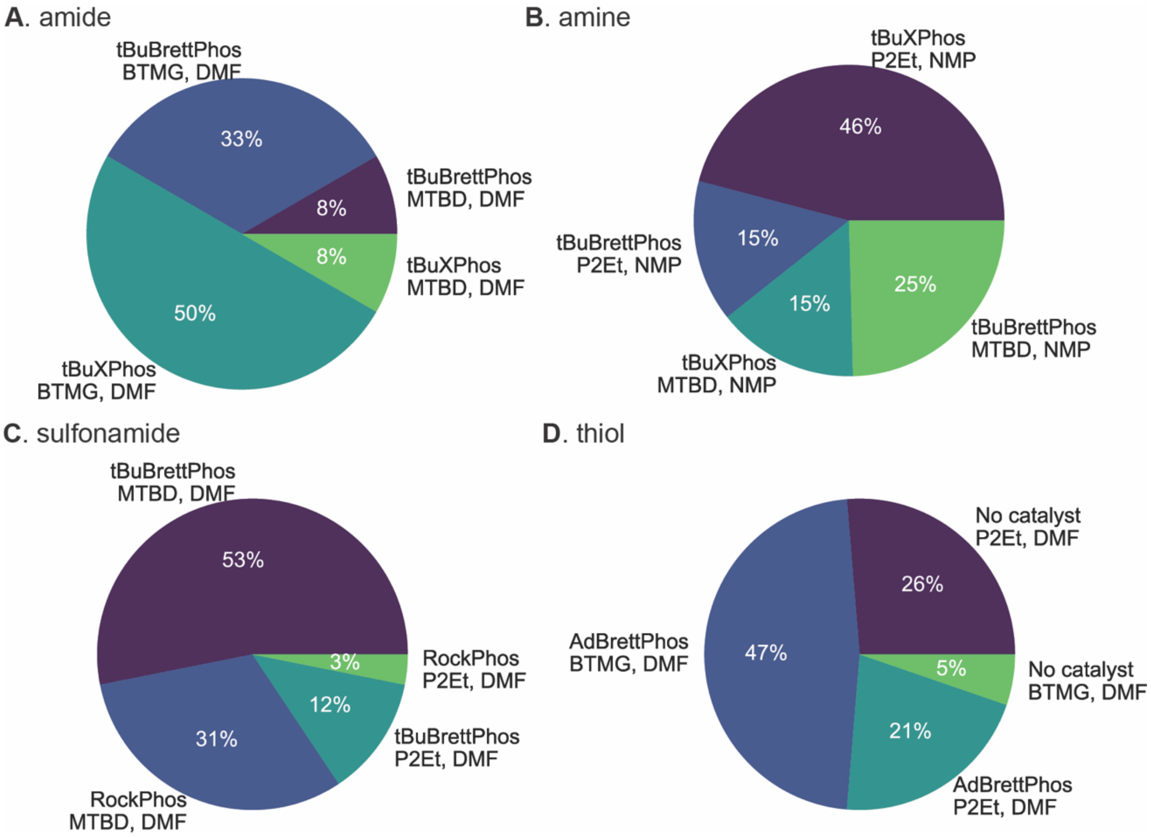

Figure S3. Portion of substrates in C–X coupling datasets that each candidate reaction condition returns highest yields after processing.

The dataset originally evaluated 32 amide nucleophiles. For the convenience of descriptor preparation, secondary amides were removed, resulting in 24 primary amide substrates. In the final form of the dataset, BTMG was the base used in the top condition for over 80% of the substrates (Figure S3A).

The amine nucleophile class originally included 96 building blocks. In this work, secondary amines were removed to make the set more amenable to descriptor preparation, leaving 61 primary amines under consideration. The number of substrates that each reaction condition works best for are relatively well spread-out. The portion of the dataset that the base P2Et is in the top condition is 61%. Conditions that use tBuXPhos as ligand comes on top in 61% of the dataset (Figure S3B).

The sulfonamide portion of the dataset with 32 substrates was used as is. Similar to the amide nucleophiles, the two reaction conditions that employ MTBD as base dominate as best condition, being the best for 84% of the substrates (Figure S3C).

The initial thiol dataset included 48 substrates. We filtered out thiols that had ties in yield. While ties are not a problem, evaluations without ties allows us to judge the differentiating ability of each ML algorithm. After all, if two of the reaction conditions give the top results, there is a 50% chance of selecting one of them out of the four possible choices. Results with the full dataset after excluding substrates where the yields are the same between all four conditions are shown below in Table S1. Removing ties removed 29 thiols (their specific composition described in subsection below), leaving 19 substrates. While the combination AdBrettPhos with BTMG is effective in 9 out of 19 substrates, two conditions that use P2Et as base are the best conditions for similar numbers of substrates (Figure S3D).

To extract physical descriptors for the nucleophiles considered here, lowest energy conformers were obtained using rdkit's `AllChem.EmbedMultipleConfs()` function.[3] Then, geometry optimization was conducted using density functional theory (DFT), at B3LYP/6-311G* level of theory through Q-Chem 5.2.[4] A total of 10 descriptors were extracted as follows. Energies of the highest occupied molecular orbital (HOMO), lowest unoccupied molecular orbital (LUMO), dipole moment, natural bond order (NBO) charge of the atom forming the new bond (S of thiol and N for other nucleophiles) and the carbon adjacent to this heteroatom were collected. The optimized geometry was further processed with the python package MORFEUS[5] to compute buried volume around the heteroatom, L, B1 and B5 sterimol parameters[6] from the X–C bond and its length. Descriptors of bases and catalysts, which are only used by regressors, were used as is from a previous study.[7]

Evaluation on thiol dataset after removing substrates that have same yield values for all four reaction conditions

In the original reaction dataset of 48 thiols described above, a significant number of substrates had two or more reaction conditions that resulted in the same yield values. Specifically, 7 thiol substrates had same yields for all four reaction conditions. In addition, 17 out of the remaining 41 substrates had either two or three reaction conditions yielding

the same value at the top. Lastly, there also were 5 substrates had ties between conditions that were not the best. The MRR scores achieved by each RF-based algorithm on the original dataset that includes these 29 substrates with ties are shown and compared against that on the processed dataset without these substrates below in Table S1. With over half of the dataset having ties, even if different models recommend different reaction conditions, in many cases they will obtain the same reciprocal rank score. This is supported by the fact that the difference between the maximum and minimum score is the smallest, compared to results from all datasets in Figure 4A. The lower scores when ties are included is due to MRR being computed as the reciprocal of the average rank of ties (e.g., if the top two are ties, then MRR = 1 / ((1 + 2) / 2) = 0.667, instead of 1).

Table S1. MRR scores of each algorithm on the thiol dataset when 29 substrates with ties are included and excluded.

MRR	**Baseline**	**RFR**	**RFC**	**LRRF**	**RPC**
Ties included	0.553	0.534	0.530	0.557	0.567
Ties excluded (row 9 in Figure 4A)	0.636	0.636	0.614	0.724	0.618

1.1.3 C–N bond formation under four distinct (photo)catalytic conditions (Figures 3C, 3D)

Both datasets – one that couples 4-phenylpiperidine (**4**) with 192 electrophiles and the other that couples 192 nucleophiles with 3-bromo-5-phenylpyridine (**8**) – were processed as follows. First, substrates where all four reaction conditions failed to give product were removed. Then, substrates provided with an invalid smiles string, such as those with generic R groups or left empty were removed. As a result, 161 electrophiles coupling with **4** remained, while 171 nucleophiles were left in the dataset coupling with **8**.

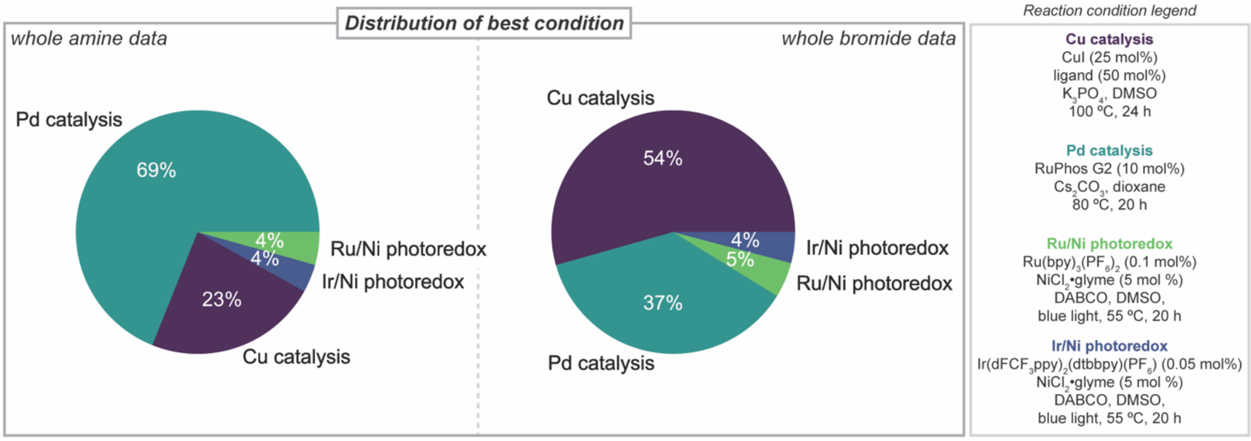

Figure S4. Distribution of each reaction condition being the highest yielding for substrates in the whole amine (left) and whole bromide (right) datasets.

Non-photoredox conditions gave the highest yields for most substrates in both datasets, accounting for over 90% the cases. Specifically, when the electrophile was fixed (whole amine dataset; left of Figure S4), Pd catalysts were dominantly the best. This is presumed to have contributed to the baseline's high MRR score 0.768 on this dataset. On the other hand, when the nucleophile was fixed (whole bromide dataset; right of Figure S4), substrate preference of Cu over Pd was more subtle.

The same set of 10 DFT descriptors were extracted from substrates as described above (last paragraph of Section 1.1.2). The reaction conditions, which are only used by regressors, were one-hot encoded due to the qualitative differences in number and form of catalysts as well as time and temperature, across the four (reaction condition legend in Figure S4).

1.2 Additional studies with fully combinatorial datasets with four reaction conditions

1.2.1 Description of the evaluation setup

Evaluations were conducted through CV. To simulate the practical situation of making predictions for new substrates, substrate splits were used. For the deoxyfluorination dataset and the C–X coupling datasets, due to the small number of substrates, they were split into 5 groups. In comparison, the two C–N coupling datasets with over 150 substrates were split into 4. Specifically, the split was determined by using scikit-learn's[15]

`StratifiedKFold()` function, which divided the substrates by the frequency of each reaction condition being the best performers.

To train the models, grid search CV was used. We will call this the ‘inner’ CV to differentiate it with the CV used for evaluation described above (which we will call the ‘outer’ CV). For the C–X coupling datasets, a 3-fold CV was used while 4-fold was used for other datasets. Below, the list of parameter grids are presented.

- RFR: ‘n_estimators’:[30, 100, 200], ‘max_depth’:[5, 10, None]
- RFC: ‘n_estimators’:[25, 50, 100], ‘max_depth’:[3, 5, None]
- kNN: ‘n_neighbors’:[3, 5, 10]
- LRRF: ‘n_estimators’:[25, 50, 100], ‘max_depth’:[4, 6, 8]
- RPC: ‘n_estimators’:[10, 25, 50, 100], ‘max_depth’:[2, 4, None]
- IBM and IBPL: ‘n_neighbors’:[3, 5, 10]

1.2.2 Logistic regression and random forest as base model of RPC

For RPC, technically any machine learning model that can output a predicted probability value can be used as the base model. While random forest classifiers were used as the base model in the main text, the original literature used logistic regression.[16] In our preliminary studies, using logistic regression as RPC’s base model and (concatenated) fingerprints of substrate(s) were compared to RPC with RFC as the base model with descriptors as input. In 9 out of 11 cases, using RFC as the base model returned higher MRR scores (Table S2, bold green). While a difference of up to 0.124 (amide dataset) was achieved among cases where using RFC gave a higher score, in the opposite case, the maximum difference was 0.057 (sulfonamide dataset). Therefore, RFC was used as the base model of RPC throughout the study.

Table S2 .Comparison of MRR scores of RPC based on logistic regression with Morgan fingerprints as input to using RFC as base model with physical descriptors as input. Bold green colors show the higher score in each dataset.

Dataset / Base model	**Logistic regression**	**RFC**
Sulfonyl Fluoride 1	0.598	**0.639**
Sulfonyl Fluoride 2	0.702	**0.734**
Sulfonyl Fluoride 3	0.653	**0.750**
Sulfonyl Fluoride 4	0.678	**0.679**

Sulfonyl Fluoride 5	0.714	**0.725**
Amide	0.600	**0.724**
Amine	0.691	**0.760**
Sulfonamide	**0.769**	0.712
Thiol	**0.627**	0.618
Whole amine	0.731	**0.760**
Whole bromide	0.672	**0.719**

1.2.3 Performance measured with top-1 accuracy

Top-1 accuracy measures the portion of cases where the highest yielding condition is ranked first by the model. As it is a practically relevant metric, model performances measured by this score is shown in Figure S5. Due to the dominance of selecting the highest yielding condition on the MRR score, observations made from relative performances in MRR scores (Figure 4A) largely hold for top-1 accuracy scores.

Datasets \ Models	Baseline	RFR	RFC	KNN	LRRF	RPC	IBM	IBPL
Sulfonyl Fluoride 1	0.375	**0.469**	**0.438**	0.344	0.406	**0.438**	0.375	0.312
Sulfonyl Fluoride 2	0.250	0.344	0.438	0.312	**0.500**	**0.531**	0.312	0.344
Sulfonyl Fluoride 3	0.406	0.344	**0.562**	0.438	0.500	**0.594**	0.406	0.375
Sulfonyl Fluoride 4	0.250	0.281	**0.469**	0.406	**0.438**	0.375	0.281	0.375
Sulfonyl Fluoride 5	**0.594**	0.531	**0.562**	0.531	0.469	0.531	0.469	0.344
Amide	0.459	**0.541**	0.492	0.459	**0.525**	0.508	0.377	0.295
Amine	0.417	**0.500**	**0.500**	0.458	0.458	**0.500**	**0.500**	0.333
Sulfonamide	0.531	**0.656**	0.562	0.438	**0.625**	0.500	0.469	0.438
Thiol	0.368	0.368	0.368	**0.421**	**0.526**	0.316	0.316	0.263
Whole amine	0.689	0.696	0.696	**0.733**	0.702	0.665	**0.708**	0.658
Whole bromide	0.409	0.532	**0.602**	0.550	**0.614**	0.561	**0.602**	0.550
Average rank across datasets	5.7	4.0	**2.8**	4.7	**2.9**	3.7	5.2	7.0

Figure S 5. Performance of each model for ranking the four reaction conditions measured by the top-1 accuracy. Green and bold black numbers correspond to the top and second-best performants in each dataset, respectively.

1.2.4 Performance measured with Kendall-tau ranking coefficient

Kendall-tau coefficient is a statistic that measures how similar two ranks are for the set of same objects (reaction conditions).[17] Specifically, for all pairs of reaction conditions, relative ranks in the actual and predicted are compared. If the preference is the same –

for example, if actual ranks and predicted ranks for two reaction conditions are (2,4) and (1, 5), respectively, the first reaction condition is preferred over the second in both – they are considered ‘concordant’. In the opposite case, the two ranks are ‘discordant’. Kendall-tau is computed as the difference in the portion of concordant and discordant pairs.

Although the quality of the whole rank between all reaction conditions is often unnecessary in the practice of organic synthesis, it is a valid way of measuring the quality of models’ recommendations of reaction conditions. Therefore, model performances measured by Kendall-tau coefficient is shown in Figure S6.

The most significant difference from using MRR as the metric is RFC’s poor performance, outperforming the baseline only in 5 out of the 11 cases. This suggests that the predicted probability of each reaction condition to be the best from RFC correlates poorly with their ranks. RFR does a better job overall since yield prediction is inherently relevant to ranking them. However, both LRRF and RPC outperforms them, appearing in the top-2 models in 10 and 8 out of 11 datasets, respectively. Altogether, this supports the importance of using models that are directly relevant to the problem of interest.

Datasets	Baseline	RFR	RFC	KNN	LRRF	RPC	IBM	IBPL
Sulfonyl Fluoride 1	0.459	**0.553**	0.363	0.376	**0.538**	0.527	0.458	0.447
Sulfonyl Fluoride 2	0.096	0.370	0.308	0.283	**0.418**	**0.547**	0.327	0.338
Sulfonyl Fluoride 3	0.385	0.436	0.398	0.414	**0.474**	**0.500**	0.463	0.369
Sulfonyl Fluoride 4	0.284	0.393	0.413	0.251	**0.504**	**0.465**	0.268	0.257
Sulfonyl Fluoride 5	0.517	0.536	0.456	0.426	**0.538**	**0.550**	0.465	0.401
Amide	0.385	0.371	0.382	0.319	0.383	**0.500**	**0.442**	0.388
Amine	0.307	0.548	0.483	0.443	**0.564**	**0.605**	0.383	0.295
Sulfonamide	0.508	**0.582**	0.407	0.413	**0.573**	0.509	0.488	0.456
Thiol	0.474	0.491	0.298	0.338	**0.579**	**0.561**	0.491	0.456
Whole amine	0.451	0.469	0.432	0.458	**0.475**	**0.475**	0.436	0.409
Whole bromide	0.401	0.448	0.481	0.474	**0.492**	0.462	**0.496**	0.464
Average rank across datasets	5.5	3.6	5.9	6.3	**2.0**	**2.1**	4.3	6.3

Figure S 6 Performance of each model for ranking the four reaction conditions measured by the Kendall-tau ranking coefficient. Green and bold black numbers correspond to the top and second-best performants in each dataset, respectively.

1.2.5 Adversarial controls with one-hot encoding and random descriptors

Kendall-tau scores of random forest (RF)-based models are higher when DFT descriptors compared to the adversarial controls of using one-hot encodings or random

descriptors (Figure S7). This is further supported by t-tests, where p-values are below 0.01 in most cases (Table S3).

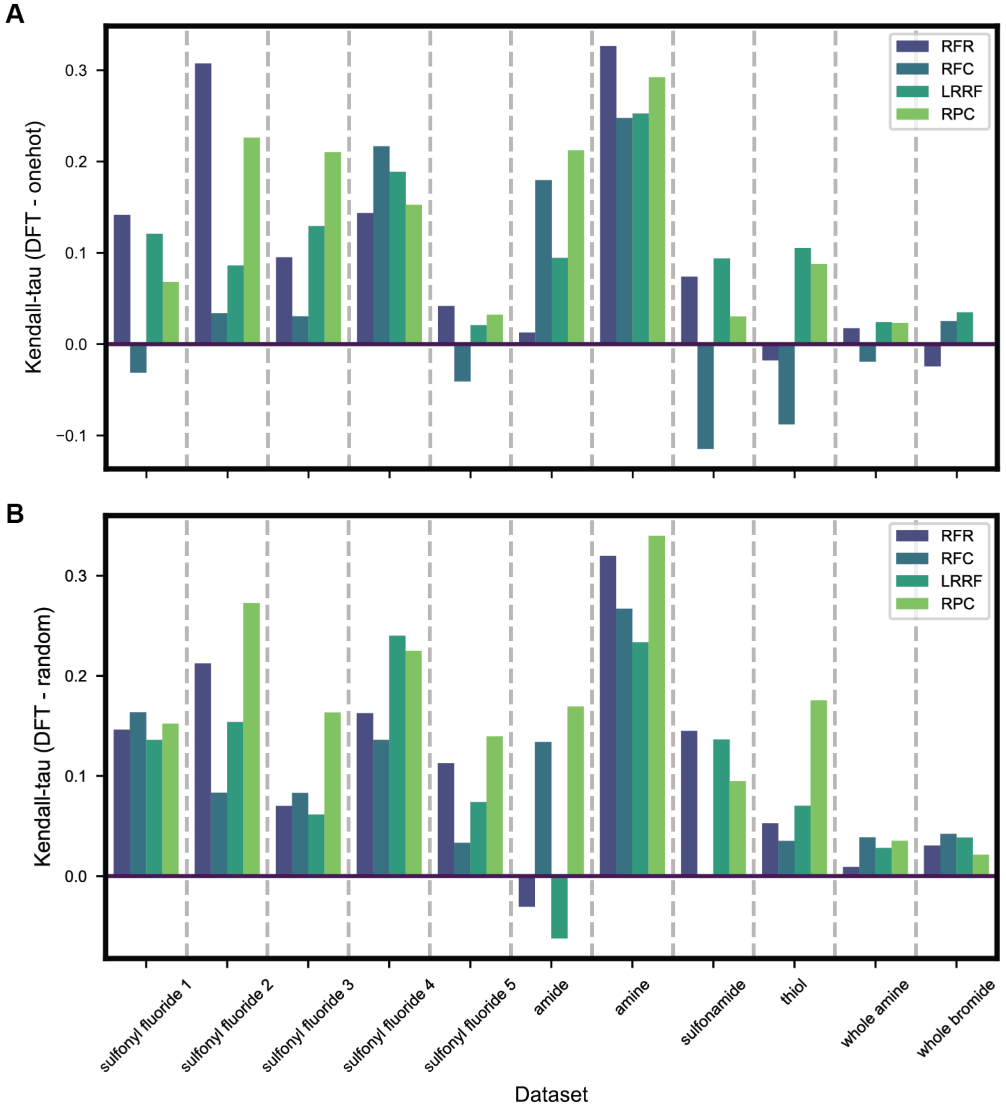

Figure S 7. Benefit of using DFT descriptors compared to when (A) one-hot encodings and (B) random descriptors are used, measured in Kendall-tau ranking coefficient.

Table S3. P-values from pairwise t-tests of Kendall-tau scores of DFT descriptors against both adversarial controls for all RF-based algorithms.

	vs. one-hot	vs. random
RFR	0.025	0.005
RFC	0.165	0.006

LRRF	0.002	0.002
RPC	3.5×10^{-4}	1.5×10^{-6}

1.2.6 Comparison of individual predictions of RF-based models

In this section, a trellis of plots analogous to Figures 4B~4E are shown for all datasets comparing all pairs of RF-based models.

Each heatmap in the right top portion of the trellis compares the rankings of reaction conditions selected by two models. Values in the diagonal (top left to bottom right) correspond to the number of cases where the two models recommended the same reaction condition. Below the diagonal corresponds to cases where the model labeled on the left predicted better conditions than the model labeled on the top (and vice versa).

The left bottom portion of the trellis shows yield differences between each pair of models. Green and blue bars correspond to substrates where the model labeled on the left predicted better than the model labeled on top and vice versa, respectively.

The values along the diagonal of the trellis correspond to the MRR scores shown in Figure 4A.

Deoxyfluorination dataset

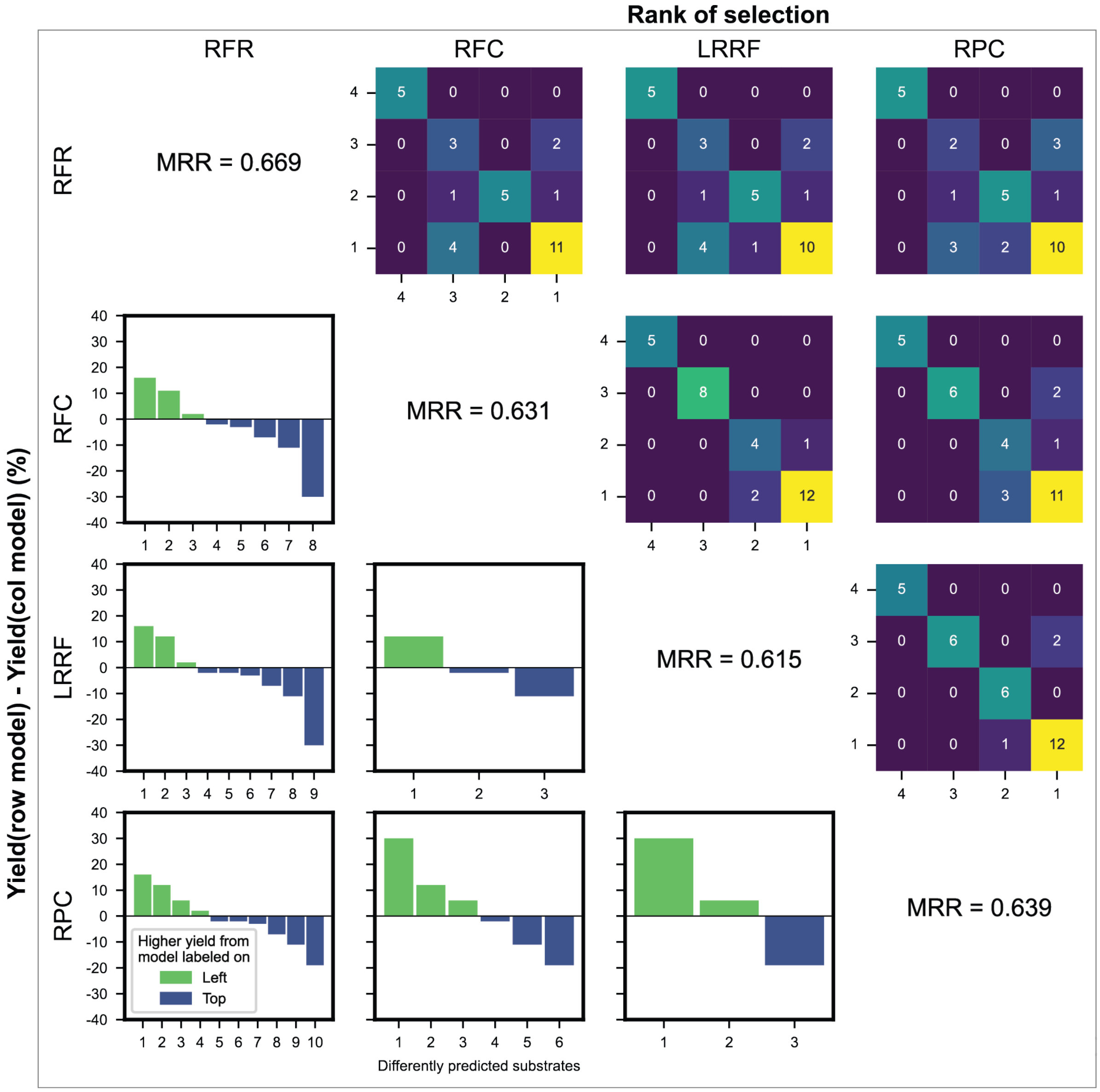

Figure S 8. Trellis of (above diagonal) heatmaps of quality of predictions measured by rank and (below diagonal) yield differences when different reaction conditions are suggested for all pairs of RF-based models in the sulfonyl fluoride 1 portion of the deoxyfluorination dataset. Green and blue bars correspond to substrates where the model labeled on the left predicted better than the model labeled on top and vice versa, respectively. Diagonal values correspond to the MRR scores of each model as shown in Figure 4A row 1.

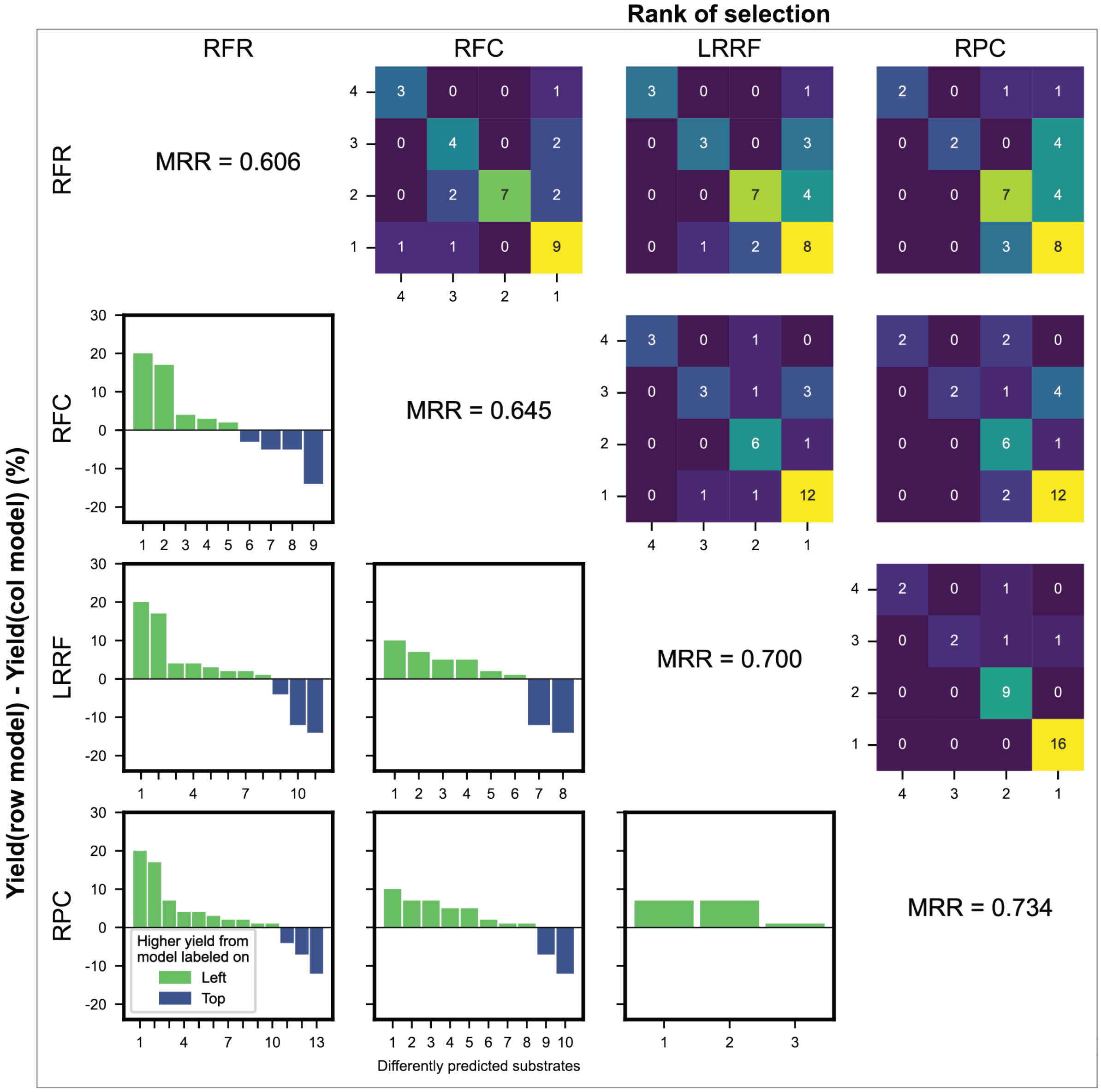

Figure S 9. Trellis of (above diagonal) heatmaps of quality of predictions measured by rank and (below diagonal) yield differences when different reaction conditions are suggested for all pairs of RF-based models in the sulfonyl fluoride 2 portion of the deoxyfluorination dataset. Green and blue bars correspond to substrates where the model labeled on the left predicted better than the model labeled on top and vice versa, respectively. Diagonal values correspond to the MRR scores of each model as shown in Figure 4A row 2.

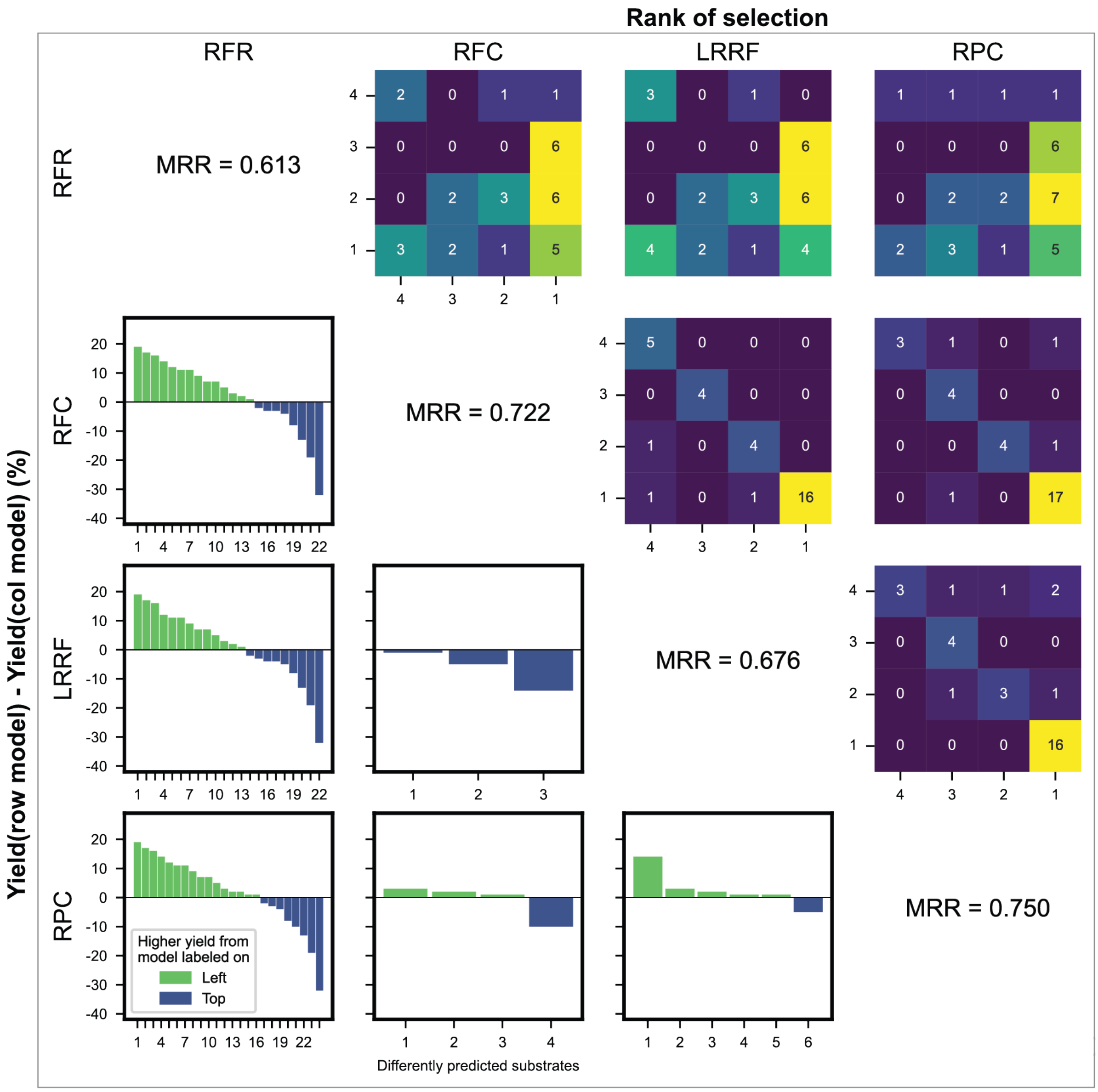

Figure S 10. Trellis of (above diagonal) heatmaps of quality of predictions measured by rank and (below diagonal) yield differences when different reaction conditions are suggested for all pairs of RF-based models in the sulfonyl fluoride 3 portion of the deoxyfluorination dataset. Green and blue bars correspond to substrates where the model labeled on the left predicted better than the model labeled on top and vice versa, respectively. Diagonal values correspond to the MRR scores of each model as shown in Figure 4A row 3.

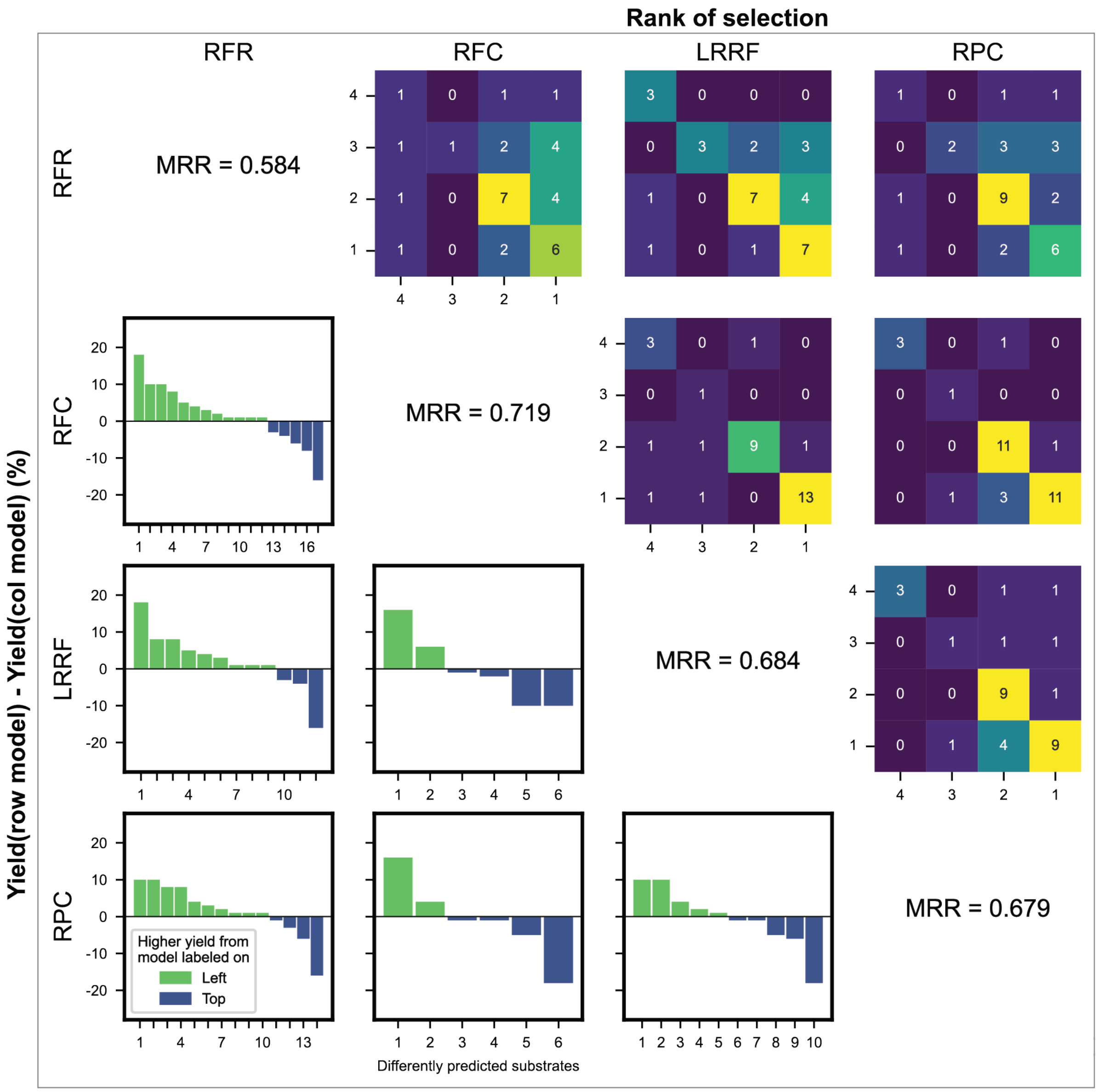

Figure S 11. Trellis of (above diagonal) heatmaps of quality of predictions measured by rank and (below diagonal) yield differences when different reaction conditions are suggested for all pairs of RF-based models in the sulfonyl fluoride 4 portion of the deoxyfluorination dataset. Green and blue bars correspond to substrates where the model labeled on the left predicted better than the model labeled on top and vice versa, respectively. Diagonal values correspond to the MRR scores of each model as shown in Figure 4A row 4.

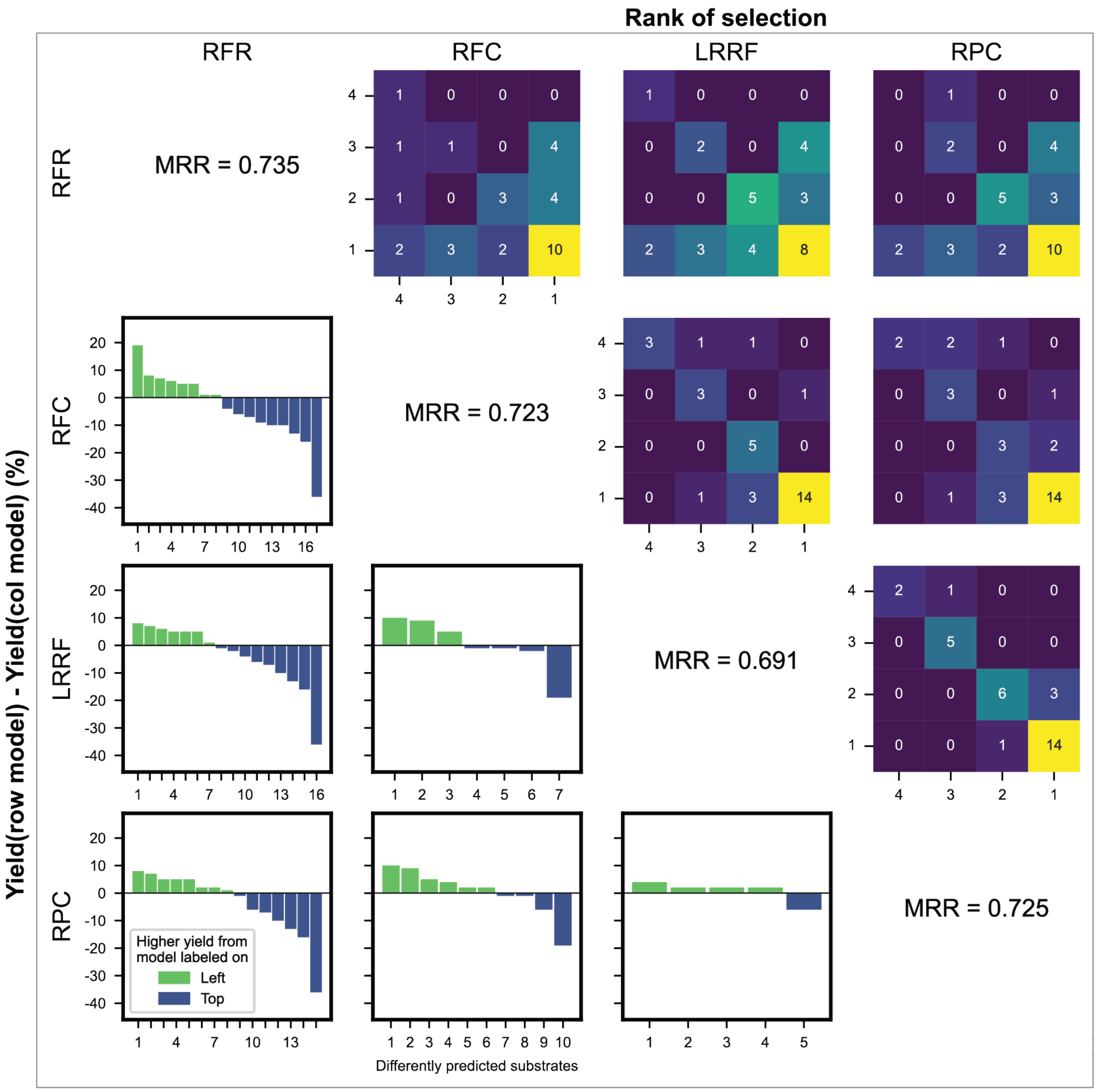

Figure S 12. Trellis of (above diagonal) heatmaps of quality of predictions measured by rank and (below diagonal) yield differences when different reaction conditions are suggested for all pairs of RF-based models in the sulfonyl fluoride 5 portion of the deoxyfluorination dataset. Green and blue bars correspond to substrates where the model labeled on the left predicted better than the model labeled on top and vice versa, respectively. Diagonal values correspond to the MRR scores of each model as shown in Figure 4A row 5.

C–X coupling datasets

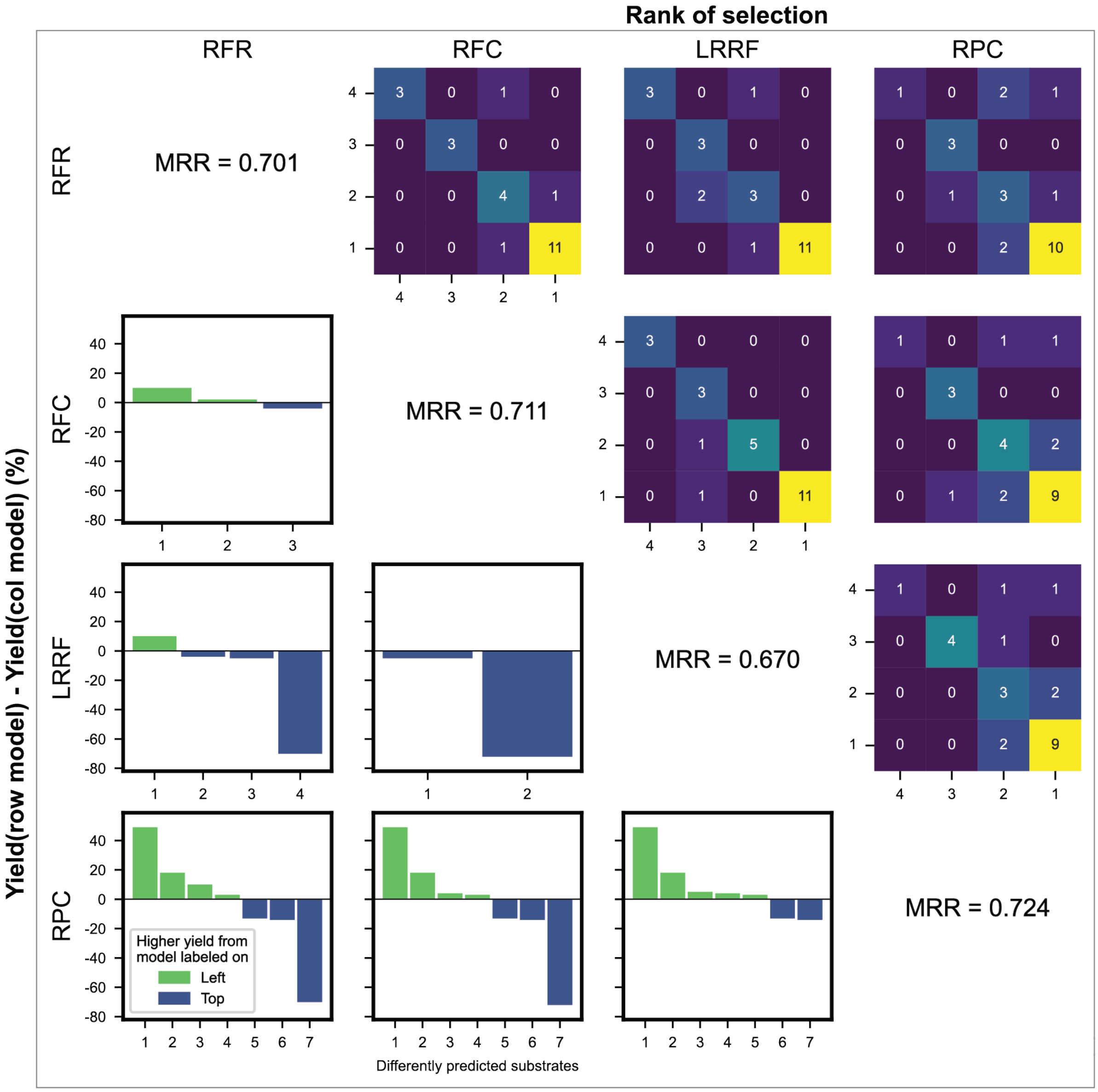

Figure S 13. Trellis of (above diagonal) heatmaps of quality of predictions measured by rank and (below diagonal) yield differences when different reaction conditions are suggested for all pairs of RF-based models in the amide dataset. Green and blue bars correspond to substrates where the model labeled on the left predicted better than the model labeled on top and vice versa, respectively. Diagonal values correspond to the MRR scores of each model as shown in Figure 4A row 6.

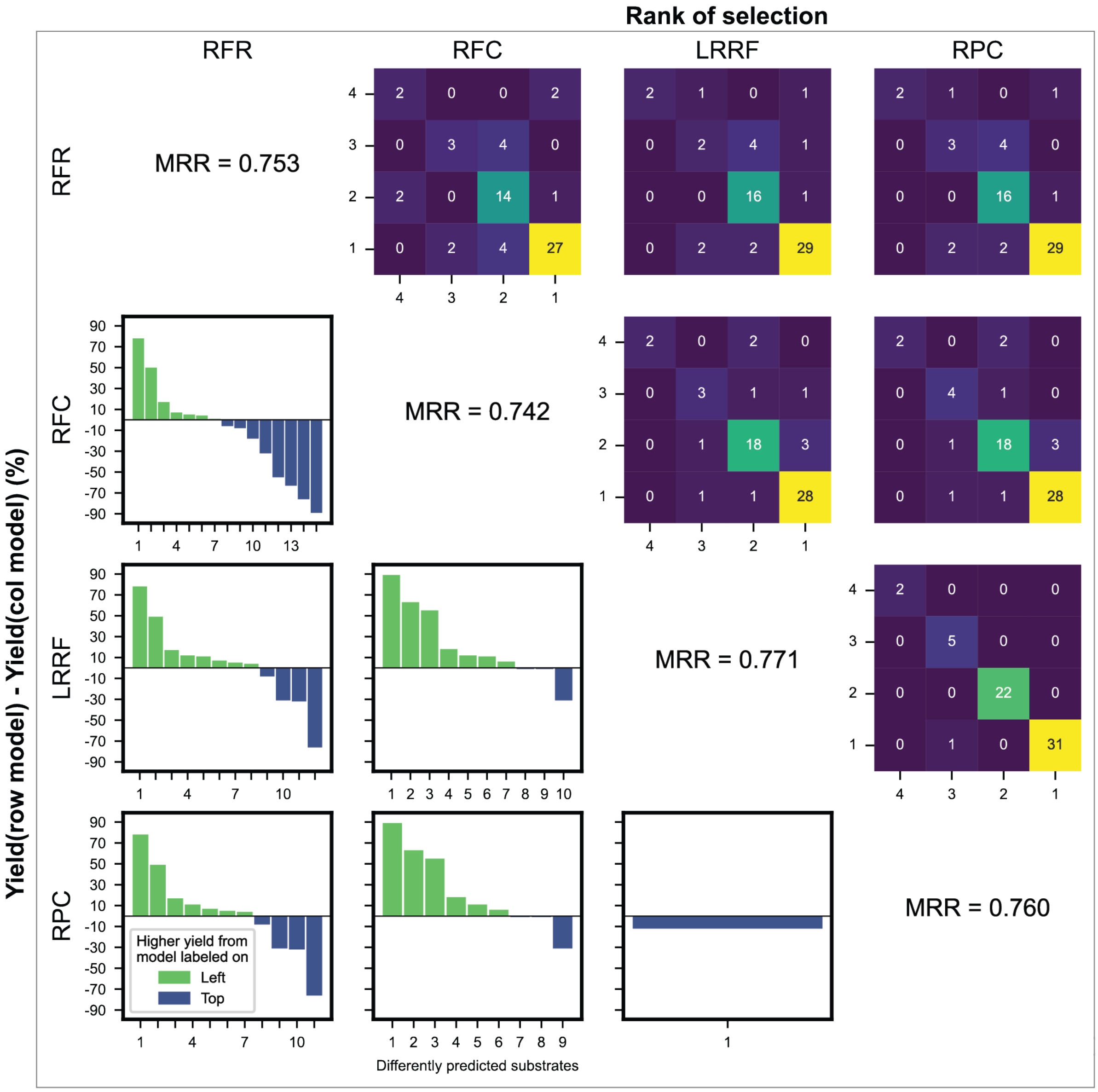

Figure S 14. Trellis of (above diagonal) heatmaps of quality of predictions measured by rank and (below diagonal) yield differences when different reaction conditions are suggested for all pairs of RF-based models in the amine dataset. Green and blue bars correspond to substrates where the model labeled on the left predicted better than the model labeled on top and vice versa, respectively. Diagonal values correspond to the MRR scores of each model as shown in Figure 4A row 7.

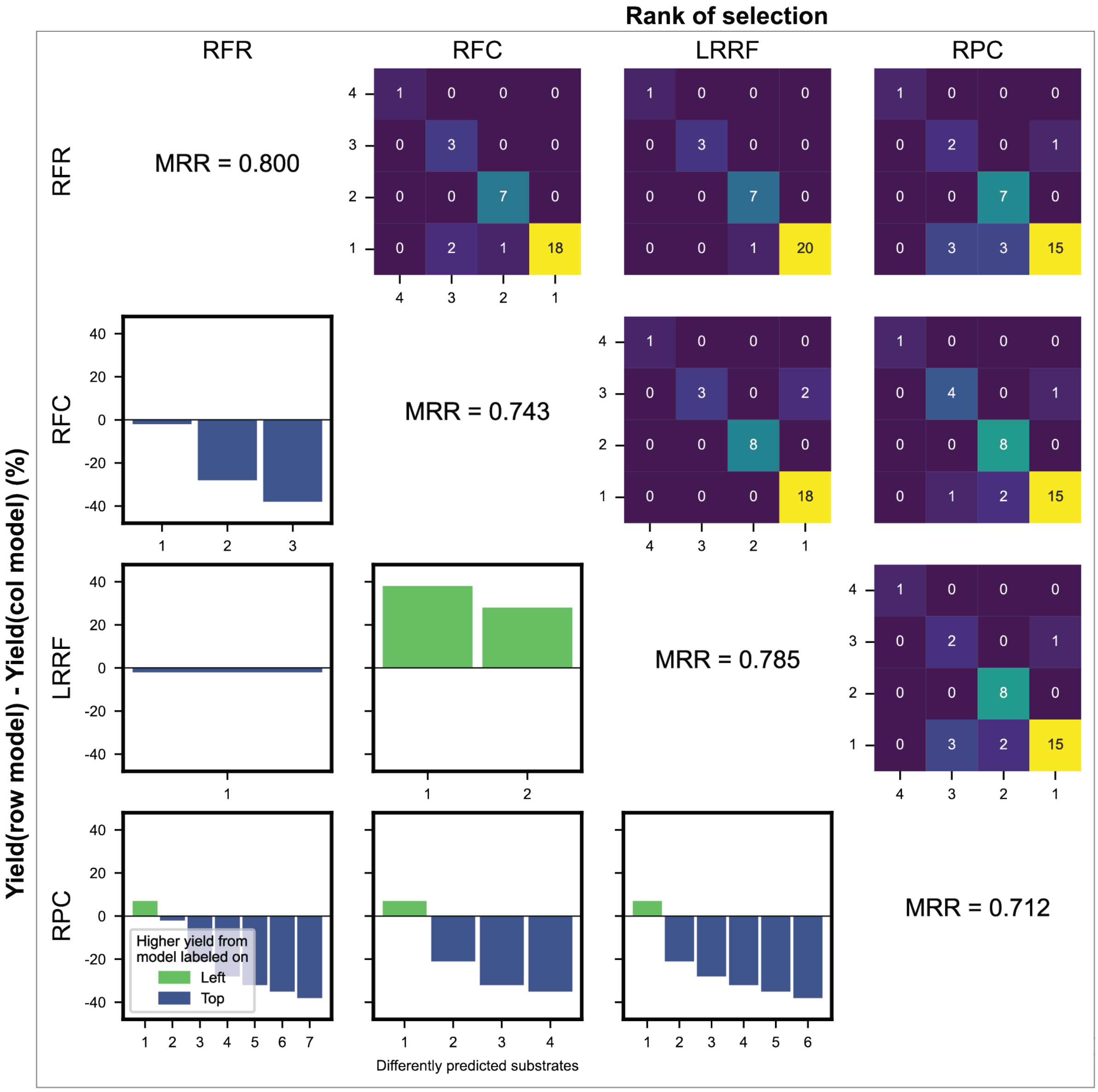

Figure S 15. Trellis of (above diagonal) heatmaps of quality of predictions measured by rank and (below diagonal) yield differences when different reaction conditions are suggested for all pairs of RF-based models in the sulfonamide dataset. Green and blue bars correspond to substrates where the model labeled on the left predicted better than the model labeled on top and vice versa, respectively. Diagonal values correspond to the MRR scores of each model as shown in Figure 4A row 8.

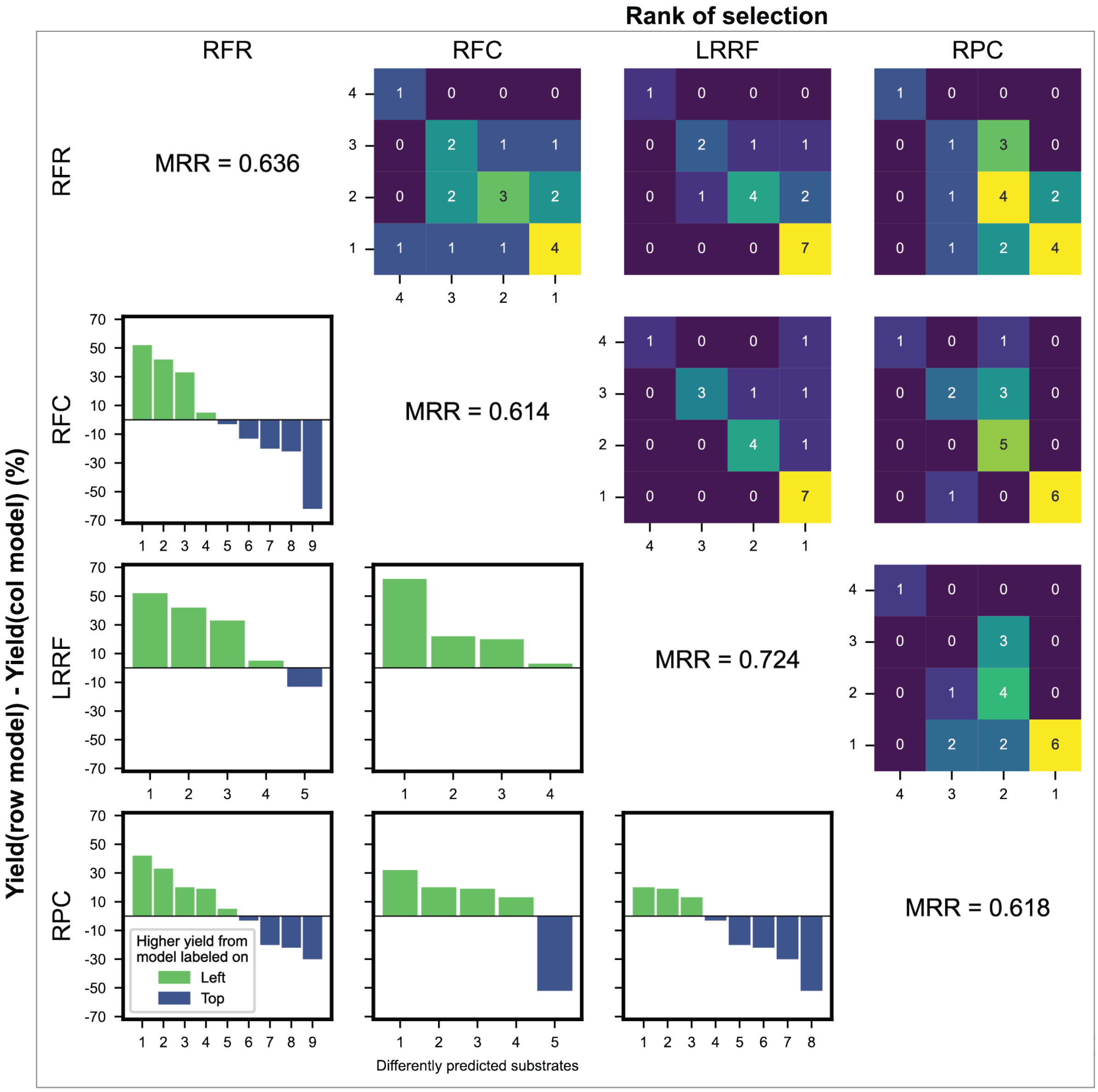

Figure S 16. Trellis of (above diagonal) heatmaps of quality of predictions measured by rank and (below diagonal) yield differences when different reaction conditions are suggested for all pairs of RF-based models in the thiol dataset. Green and blue bars correspond to substrates where the model labeled on the left predicted better than the model labeled on top and vice versa, respectively. Diagonal values correspond to the MRR scores of each model as shown in Figure 4A row 9.

C–N coupling dataset

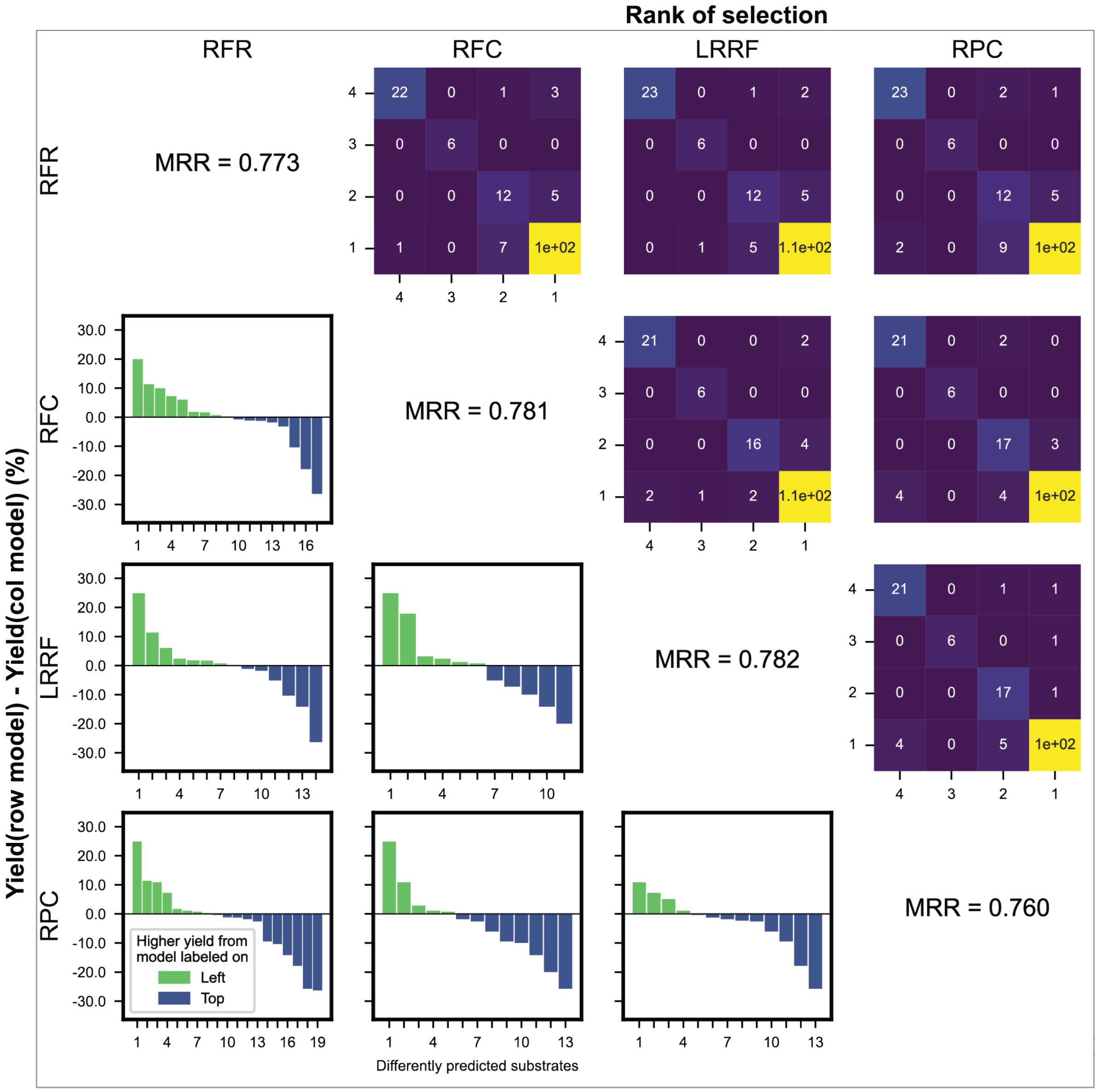

Figure S 17. Trellis of (above diagonal) heatmaps of quality of predictions measured by rank and (below diagonal) yield differences when different reaction conditions are suggested for all pairs of RF-based models in the whole amine dataset. Green and blue bars correspond to substrates where the model labeled on the left predicted better than the model labeled on top and vice versa, respectively. Diagonal values correspond to the MRR scores of each model as shown in Figure 4A row 10.

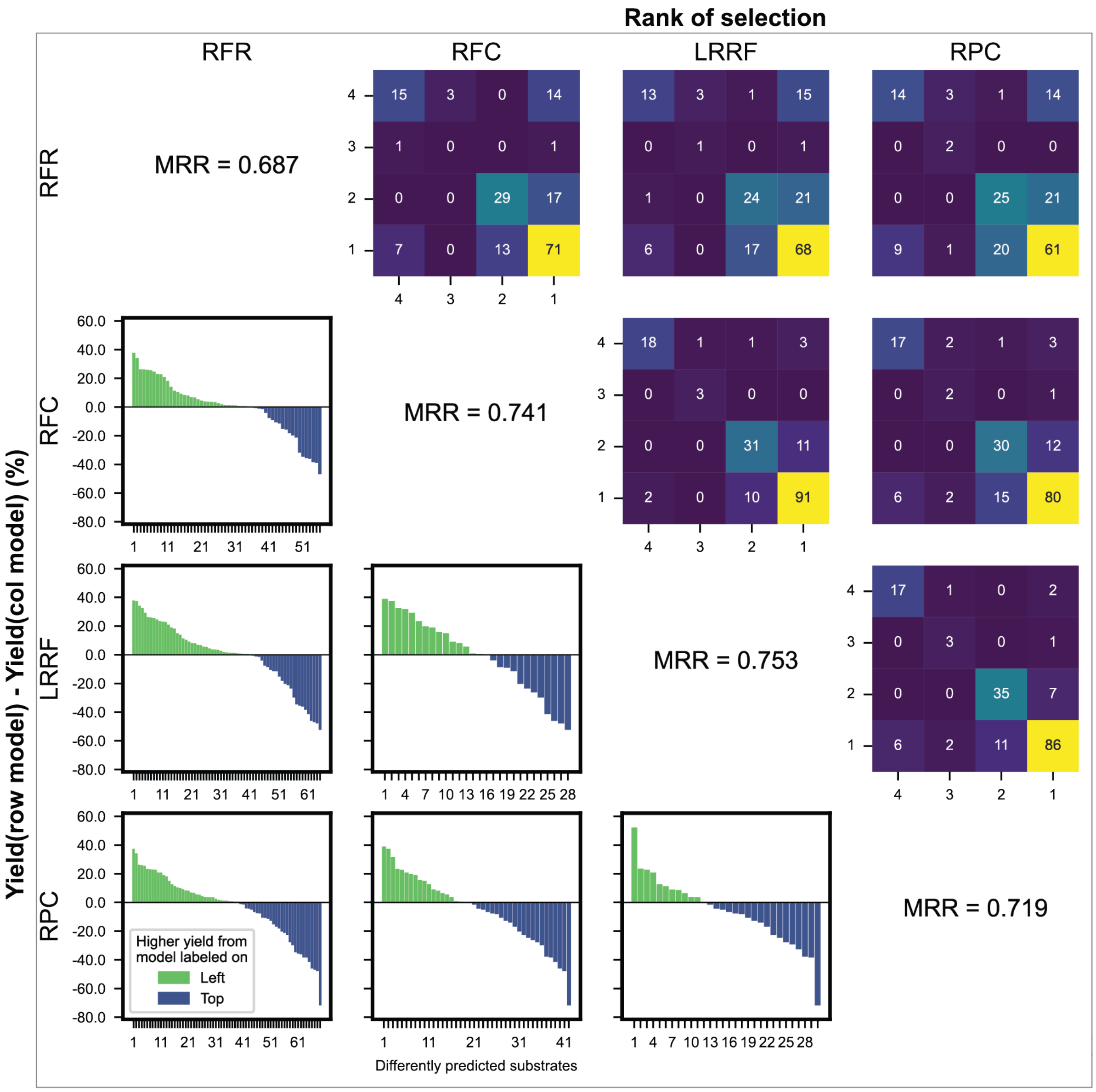

Figure S 18. Trellis of (above diagonal) heatmaps of quality of predictions measured by rank and (below diagonal) yield differences when different reaction conditions are suggested for all pairs of RF-based models in the whole bromide dataset. Green and blue bars correspond to substrates where the model labeled on the left predicted better than the model labeled on top and vice versa, respectively. Diagonal values correspond to the MRR scores of each model as shown in Figure 4A row 11.

1.3 When one of the four reaction conditions are missing

1.3.1 Description of the evaluation setup

The same CV procedure described in section 1.2.1 was used for evaluation, but with a layer of data-masking to simulate missing data. For each CV fold, outcomes of the specified number of reaction conditions are randomly erased for each substrate in the training dataset. Algorithms are trained on this masked data and evaluated on the left-out validation set. This is conducted 10 times with different sets of data-masks. The results are reported as an average across these masks and CV folds.

1.3.2 Results

Datasets	Baseline	RFR	RFC	KNN	LRRF	RPC	IBM	IBPL
Sulfonyl Fluoride 1	0.541	**0.611**	**0.601**	0.596	0.599	0.575	0.569	0.589
Sulfonyl Fluoride 2	0.465	0.578	0.605	0.552	**0.677**	**0.665**	0.560	0.568
Sulfonyl Fluoride 3	0.564	0.588	0.635	0.611	**0.664**	**0.651**	0.574	0.607
Sulfonyl Fluoride 4	0.546	0.595	0.606	0.543	**0.638**	**0.632**	0.560	0.577
Sulfonyl Fluoride 5	0.668	0.670	**0.701**	0.688	**0.702**	0.673	0.646	0.650
Amide	0.594	0.639	0.619	**0.662**	**0.666**	0.636	0.620	0.657
Amine	0.680	**0.727**	**0.725**	0.615	0.689	0.690	0.559	0.559
Sulfonamide	0.703	**0.744**	0.700	0.692	**0.722**	0.714	0.636	0.653
Thiol	0.633	0.644	0.632	0.640	**0.669**	**0.655**	0.624	0.646
Whole amine	0.768	**0.772**	0.750	0.754	0.767	**0.772**	0.693	0.691
Whole bromide	0.608	0.666	0.705	0.665	**0.724**	**0.728**	0.599	0.619
Average rank across datasets	6.4	3.2	3.9	5.0	**1.9**	**2.9**	7.1	5.6

Figure S 19. Average MRR score of each algorithm on datasets where each substrate is missing 25% of reactions. Green and bold black numbers correspond to the top and second-best performants in each dataset, respectively.

Overall, the trend in relative performance, measured by MRR, between models (Figure S19) are consistent to when fully combinatorial data are available (Figure 4A). The baseline is a poor performant. Among RF-based models, the trend of LRRF being the top-performant, followed by RPC and RFR is consistent. However, RFC is now the last in terms of average rank among the four, probably due to cases where the best reaction conditions being removed by the mask. Instance-based models showed poor performance, with IBM being worse than the baseline in average.

Datasets \ Models	Baseline	RFR	RFC	KNN	LRRF	RPC	IBM	IBPL
Sulfonyl Fluoride 1	0.303	0.369	0.372	**0.378**	**0.400**	0.338	0.338	**0.378**
Sulfonyl Fluoride 2	0.203	0.325	0.334	0.309	**0.459**	**0.428**	0.303	0.328
Sulfonyl Fluoride 3	0.319	0.338	0.400	0.366	**0.462**	**0.431**	0.316	0.372
Sulfonyl Fluoride 4	0.262	0.284	0.319	0.275	**0.375**	**0.350**	0.262	0.303
Sulfonyl Fluoride 5	0.456	0.428	**0.512**	0.488	**0.506**	0.450	0.438	0.441
Amide	0.459	**0.495**	**0.472**	0.334	0.415	0.415	0.248	0.238
Amine	0.304	0.396	0.354	**0.417**	**0.438**	0.392	0.350	0.388
Sulfonamide	0.494	**0.559**	0.481	0.469	**0.525**	0.509	0.397	0.425
Thiol	0.342	0.358	0.353	0.384	**0.411**	**0.389**	0.379	**0.389**
Whole amine	**0.689**	**0.694**	0.646	0.645	0.684	0.688	0.558	0.545
Whole bromide	0.442	0.496	0.553	0.506	**0.559**	**0.565**	0.439	0.465
Average rank across datasets	6.0	4.2	3.8	4.6	**1.9**	**3.2**	7.1	5.2

Figure S 20. Performance of each model for ranking the four reaction conditions measured by the top-1 accuracy when 25% of the data are missing for each substrate in the training data. Green and bold black numbers correspond to the top and second-best performants in each dataset, respectively.

The observations made from MRR scores largely hold for top-1 accuracy scores (Figure S20) with LRRF and RPC recording the top-2 in terms of overall rank across datasets. However, RFC is now higher in average rank than RFR. The relatively higher top-1 accuracy and lower MRR scores show that RFC can still identify the highest yielding conditions with a small portion of missing data to some degree but when it fails to do so, its utility is lower than RFR. Instance-based models showed similar trends, with kNN and IBPL being only slightly more accurate than the baseline.

Datasets	Baseline	RFR	RFC	KNN	LRRF	RPC	IBM	IBPL
Sulfonyl Fluoride 1	0.405	**0.500**	0.385	0.340	**0.450**	0.426	0.319	0.432
Sulfonyl Fluoride 2	0.134	0.342	0.367	0.235	**0.424**	**0.438**	0.258	0.270
Sulfonyl Fluoride 3	0.328	0.387	0.322	0.304	**0.448**	**0.415**	0.240	0.319
Sulfonyl Fluoride 4	0.242	0.363	0.264	0.182	**0.407**	**0.405**	0.241	0.266
Sulfonyl Fluoride 5	0.414	**0.504**	0.445	0.403	**0.498**	0.466	0.282	0.361
Amide	0.388	**0.392**	0.338	0.353	0.338	0.382	0.309	**0.408**
Amine	0.254	**0.443**	0.352	0.182	0.412	**0.423**	0.237	0.194
Sulfonamide	0.465	**0.537**	0.426	0.427	0.499	**0.505**	0.402	0.415
Thiol	0.467	0.481	0.409	0.402	**0.505**	**0.496**	0.282	0.384
Whole amine	**0.449**	**0.453**	0.391	0.400	0.440	0.446	0.308	0.326
Whole bromide	0.413	0.434	0.456	0.413	**0.461**	**0.461**	0.319	0.289
Average rank across datasets	4.7	**2.2**	4.8	6.3	2.5	**2.4**	7.5	5.6

Figure S 21. Performance of each model for ranking the four reaction conditions measured by the Kendall-tau ranking coefficient when 25% of the data are missing for each substrate in the training data.

The performance measured by Kendall-tau ranking coefficient is shown above in Figure S21. Although RFR was fourth in average rank for predicting the top reaction condition (Figure S20), the quality of the complete ranking it provides is high, being placed as the best in 5 out of 11 datasets. This is then followed by RPC and LRRF coming in a comparable average rank, despite the difference when performance was measured in MRR. This implies that RFR's ranking of reaction conditions were more accurate in the lower placements. Rankings provided by RFC, which are based on predicted probabilities of a condition being the top performant, are better than the baseline in only 5 out of 11 datasets. All instance-based models resulted in a lower average rank than the baseline.

1.4 Additional studies when two of the four reaction conditions are missing

1.4.1 Performance measured in top-1 accuracy

Datasets	Baseline	RFR	RFC	LRRF	RPC
Sulfonyl Fluoride 1	0.284	**0.338**	0.300	**0.350**	0.294
Sulfonyl Fluoride 2	0.250	0.262	0.353	**0.400**	**0.375**
Sulfonyl Fluoride 3	0.278	0.278	0.297	**0.428**	**0.381**
Sulfonyl Fluoride 4	0.250	0.247	**0.306**	**0.331**	0.284
Sulfonyl Fluoride 5	**0.425**	0.300	0.384	**0.431**	**0.425**
Amide	**0.459**	**0.444**	0.351	0.344	0.389
Amine	0.275	**0.367**	0.267	**0.338**	0.296
Sulfonamide	0.469	**0.528**	0.416	**0.472**	0.466
Thiol	0.279	0.295	**0.321**	**0.347**	0.311
Whole amine	**0.689**	**0.688**	0.553	0.679	0.659
Whole bromide	0.430	0.504	0.479	**0.512**	**0.536**
Average rank across datasets	3.6	3.0	3.6	**1.8**	**2.9**

Figure S 22. Performance of the baseline and RF-based models for ranking the four reaction conditions measured by top-1 accuracy when 50% of the data are missing for each substrate in the training data.

Similar to previous case studies, relative trends in top-1 accuracy (Figure S22) are similar to MRR scores (Figure 6A). LRRF performs the best overall being one of the top-2 performants in 9 out of 11 datasets.

1.4.2 Performance measured in Kendall-tau

Datasets	Baseline	RFR	RFC	LRRF	RPC
Sulfonyl Fluoride 1	0.381	**0.445**	0.313	**0.427**	0.407
Sulfonyl Fluoride 2	0.120	0.284	**0.295**	**0.369**	0.293
Sulfonyl Fluoride 3	0.286	**0.340**	0.223	**0.385**	0.332
Sulfonyl Fluoride 4	0.180	**0.296**	0.270	**0.302**	0.280
Sulfonyl Fluoride 5	0.339	0.423	0.318	**0.426**	**0.445**
Amide	**0.361**	0.341	0.315	**0.346**	0.332
Amine	0.257	**0.359**	0.320	**0.354**	0.298
Sulfonamide	0.466	**0.520**	0.337	0.411	**0.486**
Thiol	**0.409**	0.407	0.383	**0.412**	0.351
Whole amine	**0.431**	**0.420**	0.334	0.418	0.419
Whole bromide	0.405	**0.434**	0.373	0.431	**0.440**
Average rank across datasets	3.5	**2.2**	4.4	**2.1**	2.9

Figure S 23. Performance of the baseline and RF-based models for ranking the four reaction conditions measured by the Kendall-tau ranking coefficient when 50% of the data are missing for each substrate in the training data.

When 50% of the training reaction data were missing, LRRF was overall the best in terms of Kendall-tau score coming on top in 4 of 11 datasets (Figure S23). RFR showed similar performance showing highest scores in 3 of 11 datasets. The average rank of RPC was notably lower than these two algorithms in contrast to previous cases where it was the second best. This implies RPC's weakness under significant missing data stems from inability to correctly order the lower performing reaction conditions compared to RFR and LRRF. RFC, consistent with the previous results, is incapable of ordering the set of four reaction conditions relative to the other RF models, supporting the low correlation between predicted probability values and yields.

1.4.3 Decrease in MRR score due to incomplete data in all datasets

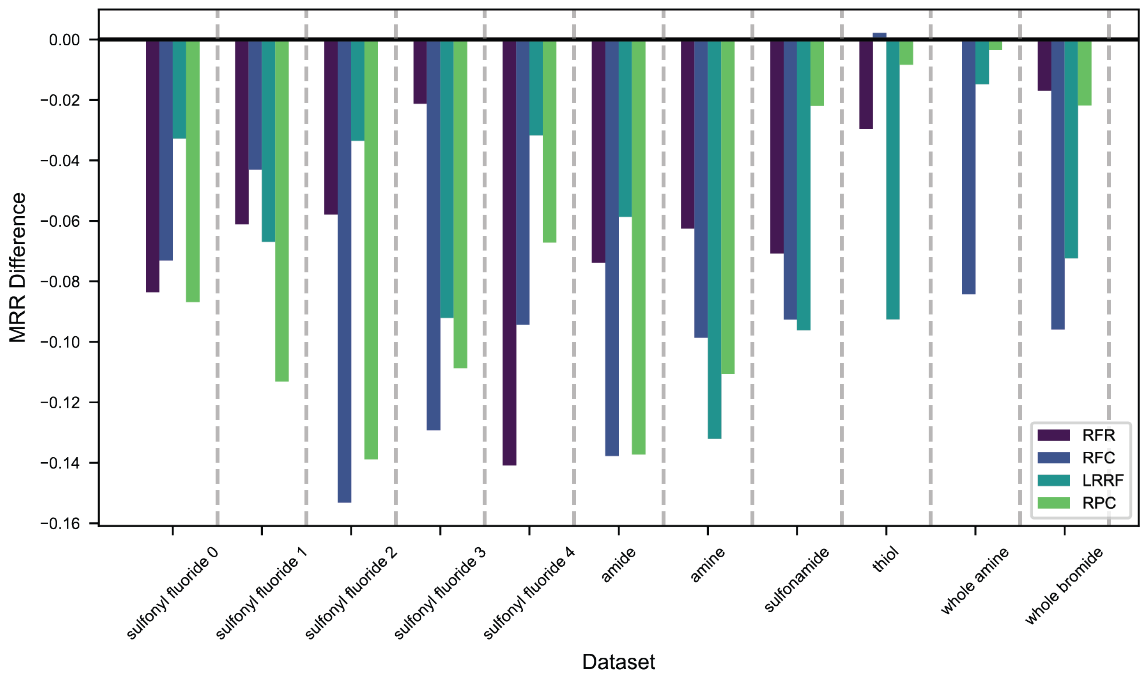

Figure S 24.Decrease in MRR of each algorithm on all datasets when 50% of the training dataset is available compared to the fully combinatorial case.

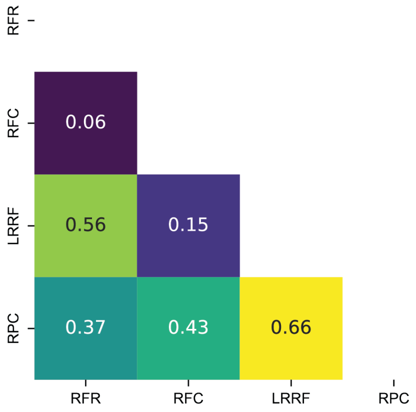

Figure S 25. P-values from t-tests of values of MRR decrease for all pairs of RF-based models.

With these evaluations in hand, the robustness of each algorithm to missing data was compared next. Figure S24 shows the decrease in MRR when 50% of the training data is masked, compared to having the full data (extension of Figure 5B). The absolute size of the dataset does not seem to correlate with the extent of the decrease in MRR, as the values in the two rightmost columns (the largest datasets with >150 substrates) are

comparable to those in other columns (19 substrates in the thiol dataset). Also, the algorithm that degrades the most in performance are different for different datasets. In all, comparison of these values by pairs of algorithms does not show statistical significance under the datasets used in this study (Figure S25).

1.4.4 Kernel density estimation of standard deviation of MRR in all evaluations with missing data

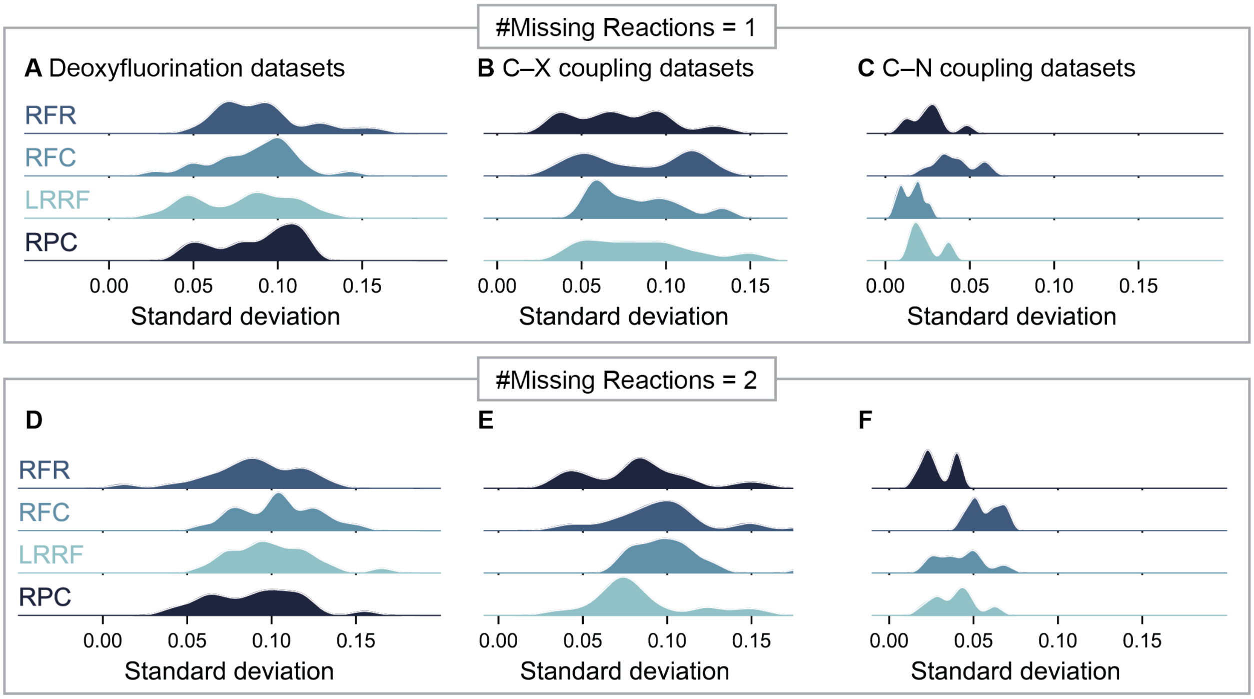

Figure S 26. Kernel density estimation plots of standard deviation of MRR scores across data masks, collected across cross-validation folds and datasets.

Figure S26 presents the Kernel density estimation plots across all datasets under different numbers of missing reactions. The distribution in the C–N coupling datasets (Figure S26C and S26F) are the narrowest among the three groups of datasets, by roughly a factor of two. This is likely due to them being the largest dataset, allowing the models to learn how relative performances between conditions are correlated to substrate features despite the missing data.

Under the same dataset group (each column in Figure S26), when the number of missing reactions increases, there is a notable shift of distribution of MRR standard deviations to wider ranges for RFC and LRRF. This can be attributed to the algorithms being vulnerable to the actual best condition being masked out. Between the LR

algorithms, when only one reaction is missing, LRRF's distribution of MRR standard deviation is narrower than RPC, while it is the opposite when two reactions are masked.

2 Datasets with a larger number of possibilities

2.1 Dataset preprocessing, featurization, and distribution of best conditions

2.1.1 Ullmann dataset (Figure 3E)

The Ullmann dataset was curated in two steps in the original report.[12] The first set of ligands were selected manually from the literature. The results showed that 6 ligands that closely bind to Cu (less than 2.07Å) were important for obtaining yields above 20%. Based on this observation, 12 more ligands were selected and the combined set of 18 ligands were evaluated against more substrate pairs. In this study, substrate pairs that have been evaluated with these 18 ligands were used for evaluating label ranking (LR). The DFT descriptors provided in the reference[12] were used as is.

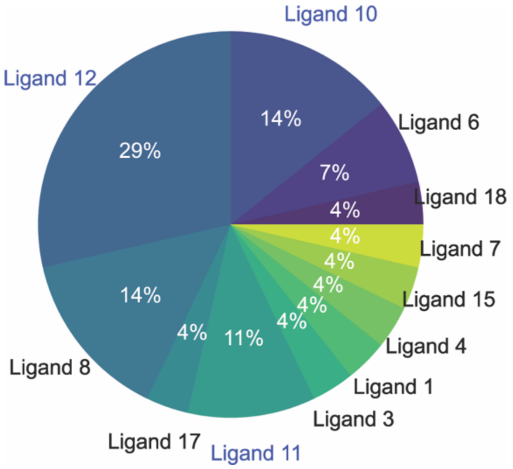

Figure S 27.Portions of each ligand being the best for the substrates in the Ullmann dataset. Ligands in the top-4 in terms of average yield are marked blue.

In agreement with the Ullmann reaction's perceived sensitivity of reaction condition to substrates, the best ligand turns out to be different for different substrates (Figure S27) and thus presents an interesting case for reaction condition prediction.

2.1.2 Nickel-photoredox dataset

Dual nickel-photoredox catalysis has shown to catalyze various useful transformations[18,19], including C–N coupling. This mode of coupling has been evaluated using HTE on complex aryl halides that are representative of drug-like molecules.

Specifically, 18 (hetero)aryl halide informers have previously been subject to a total of 96 reaction conditions to identify robust nickel-photoredox dual catalytic reaction conditions for C–N coupling.[8] The 96 conditions were all combinations between 12 iridium photocatalysts, 4 nickel:photocatalyst ratio values, 2 amine stoichiometry values.

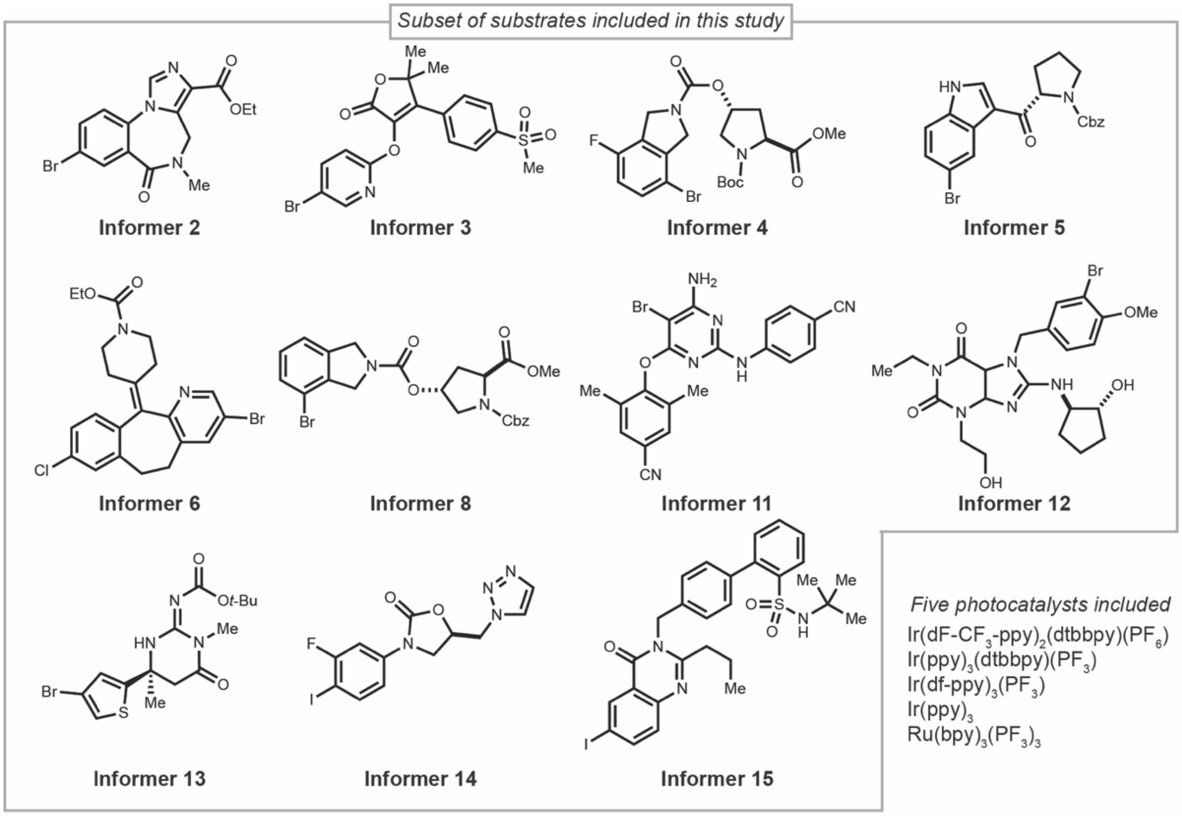

Figure S 28.Overview of the substrates and photocatalysts which are subsets taken from the original dataset, used in this study.

To process the dataset, substrates that consistently returned below 20% yield were removed, leaving 11 informers (Figure S28). Then, the photocatalysts were filtered. Among the photocatalysts, 7 of them which returned below 20% yield in 9 out of the 11 informers were also removed. Representation of the reaction components was done as follows:

• Substrates: Instead of the 10 physical descriptors listed in Section S1.1.2, 1024-bit Morgan fingerprints with radius 3 was used to capture the structural diversity.

• Photocatalysts: Three physical measures – reduction potential from their excited triplet to doublet, reduction potential from their doublet to triplet form, and maximum absorption wavelength – were collected from the literature.[9–11]

• Stoichiometry values were one-hot encoded.

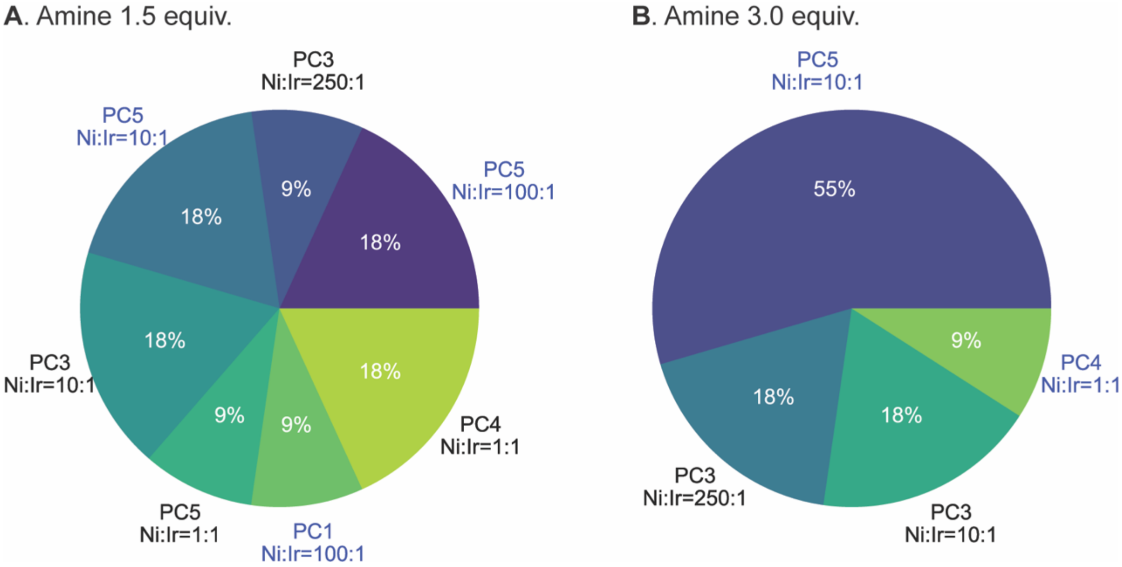

Figure S 29. Distribution of each combination of photocatalyst, catalyst ratio being the highest yielding for informers for each stoichiometry value of piperidine: (a) 1.5 equiv and (B) 3.0 equiv. Reaction conditions included in the top-4 in terms of average yields are marked blue.

When 1.5 equiv. of piperidine was used, a diverse set of reaction conditions gave the highest yields for different informers (Figure S29A). Recommending a fixed reaction condition for substrates is therefore unlikely to be effective. Among the four conditions selected by the baseline, three of them show up as a top condition (marked blue in Figure S29A) and are successful in only 45% of the cases. In contrast, when 3.0 equiv. of piperidine was used, only 4 out of 20 conditions appear at the top. Moreover, one of them was the best for 6 out of 11 substrates (Figure S29B).

2.1.3 C–H borylation dataset

Selective C–H borylation reactions are valuable as the product provides a handle to couple with other building blocks through Suzuki coupling. In the previous report,[20] to develop a model that can predict yield and regioselectivity of C–H borylation of 40 substrates, 6 ligands and 4 solvents that are commonly used for this transformation were

evaluated. Among these reagents, 2 ligands and 1 solvent that consistently returned poor yields were removed as these reagents do not show up within the top-4. This modification is reasonable since it is practical to focus on reaction conditions that are promising for future substrates after initial evaluation. This leaves a total of 12 reaction conditions to choose from. From the 40 initial substrates, those that gave at least three positive yields were kept, leaving 16 substrates.

Due to the high diversity in substrate structure and C–H bonds where the reaction occurs, instead of DFT descriptors, Morgan count fingerprints of length 1024 and radius of 3 was used for all algorithms. For the regressors, one-hot encoding was used for ligand features while five solvent parameters (dipole moment, Hansen D, P and H parameters and Abraham Vx parameter) were extracted from the ACS solvent selection tool.[13]

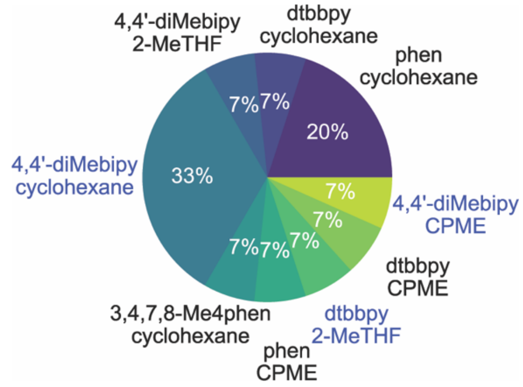

Figure S 30. Portions of substrates each reaction condition was the highest yielding. The three ligand, solvent combinations that return the highest average yields are marked blue.

Consistent with other datasets that screened more than 10 reaction conditions, the C–H borylation dataset also show different reaction conditions being the most effective for different substrates (Figure S30).

2.1.4 Aryl halide borylation dataset

Nickel-catalyzed borylation of aryl (pseudo)halides presents another way to prepare valuable cross-coupling partners while also amenable to subsequent Suzuki coupling in the same pot. To further understand this important reaction, a previous report evaluated this reaction on 33 diverse substrates using 23 different ligands.[14] In the file provided by the authors, three reaction datapoints were missing. By filling these missing datapoints

with 0% yield, the dataset became a fully combinatorial grid of 33 substrates reacting with 23 ligands. Provided physical descriptors of all reaction components were used as is.[14]

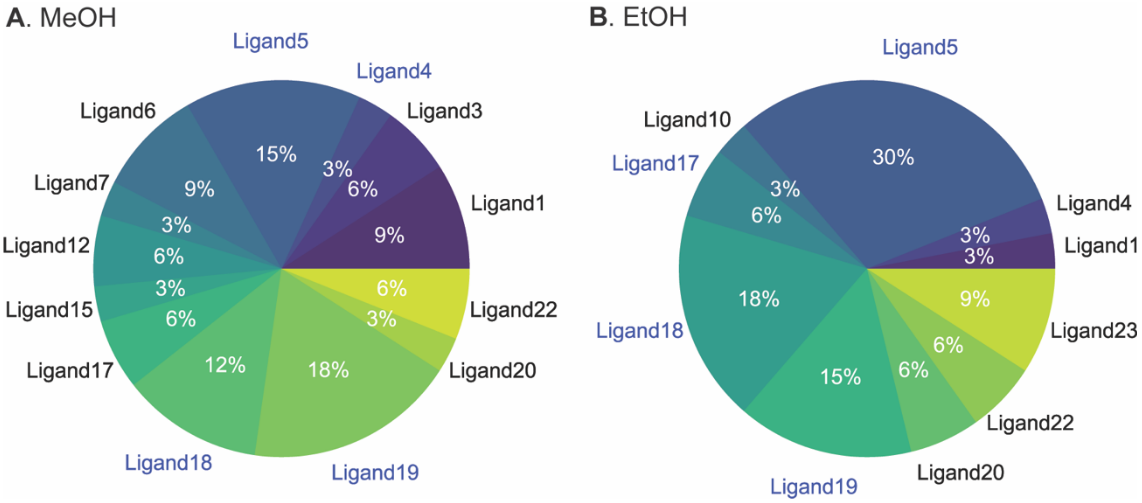

Figure S 31. Portions of substrates each ligand was the highest yielding. Four ligands that give the highest average yields are marked blue.

The distribution of the best ligand for each substrate was wide for both solvents (Figure S31). For both solvents, ligands 5, 18 and 19 are those that gave highest average yield across all substrates. They turn out to be the best ligand with the highest frequencies, accounting for 45% and 63% of the substrates in MeOH and EtOH datasets, respectively (portions of Figure S31 for ligands marked with blue).

2.2 Evaluation setup

Like the evaluations of algorithms on datasets with four reaction conditions, CV was used for datasets with a larger number of possibilities. The grid of parameters used for each model was the same as before (section 1.2.1). The CV-folds used for each dataset were defined as below.

Table S 4. CV splits used for both screening through parameter combinations and evaluations in each dataset and the number of reaction conditions selected by each algorithm.

**Dataset**	**Inner-CV (parameter screen)**	**Outer-CV (evaluation)**	**Number of selections**
Ullmann	4	4	4
C–H borylation	4	5	3
Nickel-photoredox	Leave-one-out	Leave-one-out	4
Ar–X borylation	4	5	4

2.3 Results

2.3.1 Performance measured in reciprocal rank of the best selected condition

Datasets \ Models	Baseline	RFR	RFC	LRRF	RPC
C-H Borylation	0.589	0.563	0.587	**0.616**	0.651
Nickel-Photoredox (1.5 equiv. amine)	0.582	0.451	0.605	**0.582**	0.514
Nickel-Photoredox (3.0 equiv. amine)	**0.624**	0.568	0.670	0.600	0.600
Aryl Halide Borylation in EtOH	0.838	0.580	0.680	**0.785**	0.763
Aryl Halide Borylation in MeOH	0.651	0.448	0.594	**0.610**	0.598
Ullmann	0.604	0.608	0.728	0.780	**0.773**
Average rank across datasets	**2.4**	4.8	2.8	2.2	2.8

Figure S 32. Performance of each model measured by the reciprocal rank of the best among the multiple selected conditions on datasets with more than four reaction conditions to choose from. Green and bold black numbers correspond to the top and second-best performants in each dataset, respectively.

2.3.2 Performance measured in average reciprocal rank of all selected conditions

Datasets \ Models	Baseline	RFR	RFC	LRRF	RPC
C-H Borylation	0.303	0.285	**0.311**	0.308	0.319
Nickel-Photoredox (1.5 equiv. amine)	**0.271**	0.209	0.290	0.264	0.244
Nickel-Photoredox (3.0 equiv. amine)	**0.286**	0.262	0.292	0.269	0.282
Aryl Halide Borylation in EtOH	0.348	0.240	0.299	**0.317**	0.304
Aryl Halide Borylation in MeOH	0.281	0.215	0.256	**0.269**	0.259
Ullmann	0.261	0.282	0.313	**0.339**	0.342
Average rank across datasets	2.5	4.8	**2.5**	2.7	2.5

Figure S 33. Performance of each model measured by the average of reciprocal rank of all selected conditions on datasets with more than four reaction conditions to choose from. Green and bold black numbers correspond to the top and second-best performants in each dataset, respectively.

2.3.3 Performance measured in Kendall-tau

Datasets	Baseline	RFR	RFC	LRRF	RPC
C-H Borylation	0.161	0.166	0.130	**0.210**	**0.209**
Nickel-Photoredox (1.5 equiv. amine)	**0.312**	0.199	0.238	0.311	**0.350**
Nickel-Photoredox (3.0 equiv. amine)	0.331	0.294	0.290	**0.376**	**0.408**
Aryl Halide Borylation in EtOH	**0.514**	0.182	0.390	**0.492**	0.469
Aryl Halide Borylation in MeOH	**0.409**	0.105	0.301	**0.389**	0.384
Ullmann	0.240	0.280	0.263	**0.326**	**0.323**
Average rank across datasets	2.7	4.2	4.3	**1.8**	**2.0**

Figure S 34. Performance of each model measured by the Kendall-tau ranking coefficient on datasets with more than four reaction conditions to choose from.

2.4 Adversarial controls

Figures S35 and S36 compare the performance of models using fingerprints (C–H borylation and nickel-photoredox datasets) or DFT descriptors (aryl halide borylation and Ullmann datasets) against one-hot encodings and random descriptors (on datasets with DFT descriptors only), respectively. Most notably, in all datasets except the Ullmann dataset, the use of 'meaningful' features fails to return higher scores than the controls (which otherwise would result in bars on the positive side) across all three metrics. This might be a result of the combination of two factors.

1) Dataset structure: the number of substrates to learn from is similar to the number of reaction conditions to rank between. Also, the highest yielding reaction conditions were diverse, as we saw above in Section S2.1. Together, this makes it a challenging problem for ML algorithms.
2) Descriptors: there is a possibility that the selected descriptors do not correlate with the reactivity well. Also, the large number of features compared to the substrates make it difficult to extract truly meaningful ones that actually impact outcome.

Accordingly, only the results from the Ullmann dataset are discussed in the main text.

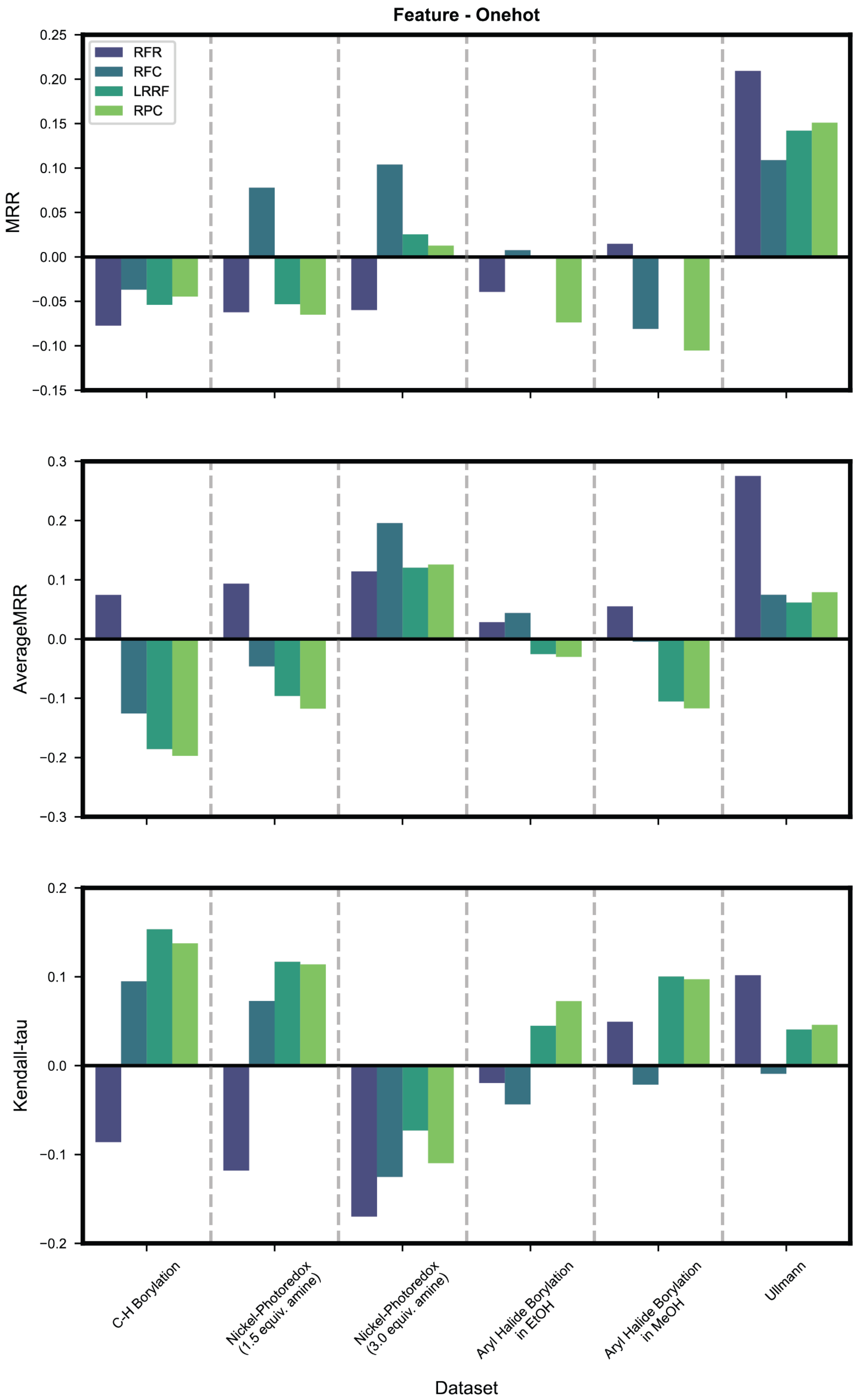

Figure S 35. Benefit of using either fingerprints (left three columns) or DFT descriptors (right three columns) compared to when one-hot encodings are used, measured in three different metrics. Bars pointing up corresponds to cases where the features scored higher.

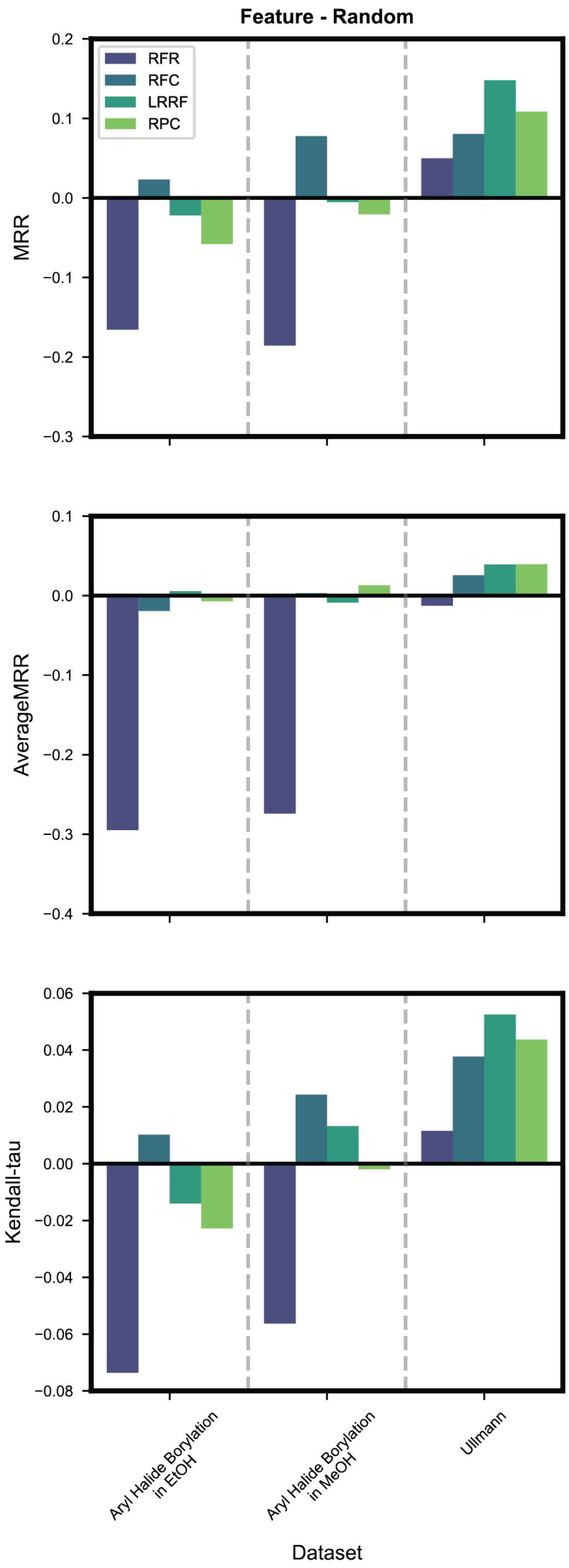

Figure S 36. Benefit of using DFT descriptors compared to when random descriptors are used, measured in three different metrics for datasets that provided the descriptors. Bars pointing up corresponds to cases where the features scored higher.

2.5 RFR's individual predictions on all CV folds of the Ullmann dataset

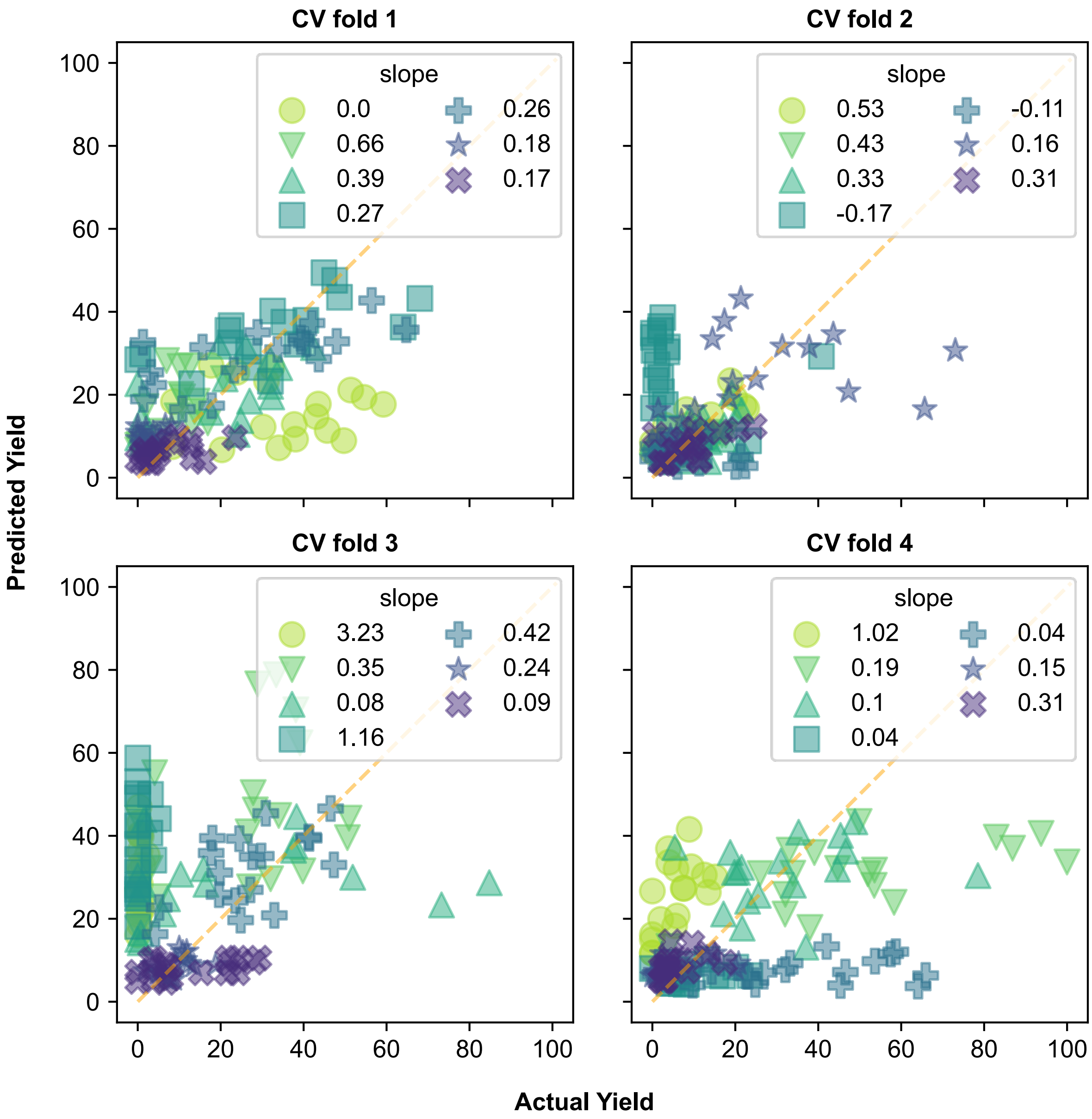

Figure S 37. Scatter plot of actual and RFR-predicted yield values in all CV splits of the Ullmann dataset. Each marker corresponds to one test substrate's results with different ligands. Slope (computed from linear regression) approximates the model's ability to differentiate ligands.

For suggesting four best ligands in the Ullmann dataset, RFR showed performance comparable to the baseline (Figure 6A). To analyze the poor performance, the predicted versus actual yield for test substrate pairs was plotted for each CV fold (Figure S37). The slope of each marker (corresponding to one substrate pair) represents ligand's impact on yield that is captured by the model. Out of a total of 28 substrates, 16 show slopes below

0.3, indicating RFR's poor differentiation between ligands. This suggests that the 21 training substrates were insufficient for RFR to learn how the 18 ligands behave with new substrates, despite the significant training dataset size of 336 reactions.

This shortcoming is present despite models' effort to learn the dependency of reaction outcome on the substrates. This effort is supported by the sum of feature importance of the substrate descriptors being larger than those of the ligand in models of all CV folds (Figure S38). Simultaneously, however, the relatively small use of ligand descriptors could have led to a difficulty in differentiating between and ranking the ligands, which is the goal of this problem.

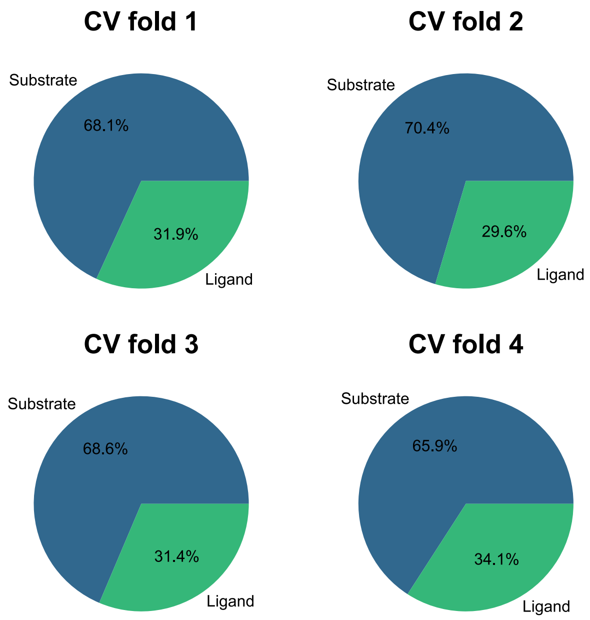

Figure S 38. Sum of feature importance values of substrate and ligand descriptors in each CV fold in the Ullmann dataset.

2.6 Comparison of RFR's and LRRF's predictions on the Ullmann dataset

Lastly, predictions from RFR and LRRF were compared. Out of all 28 substrates, the best ligand among the four suggestions from the two algorithms differed in 10 cases (Figure S39A). In 8 out of the 10 cases, LRRF included a better choice and 3 cases resulted in greater than 10% yield benefit (Figure S39A green bars). The largest yield difference was 29% favoring the prediction from LRRF (Figure 6D). In contrast, the yield

difference from the two substrate pairs where RFR gave better results was lower than 10% (Figure S39A blue bars). In Figure S39A, bars with black borders correspond to cases where one algorithm identified the top ligand while the other one could not. Such cases are observed with seven green bars, meaning LRRF was able to identify the best ligand while RFR could not. However, there was no case where RFR identified the top ligand while LRRF was not able to.

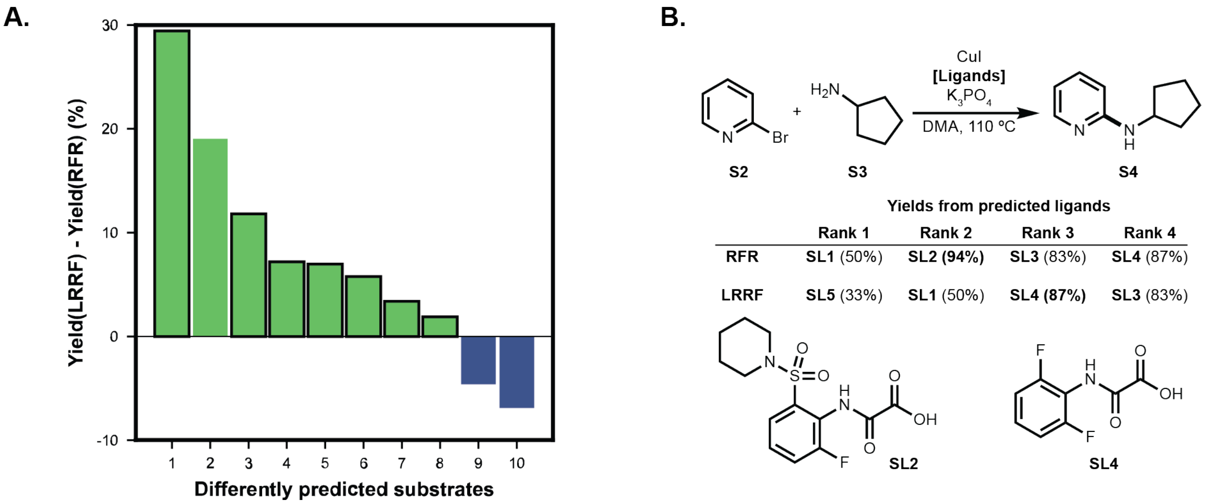

Figure S 39. (A) Yield difference for cases where the best performing ligands were differently predicted. Green and blue bars correspond to substrates where LRRF and RFR made better choices, respectively.

The specific reaction where RFR's benefit was the largest (rightmost blue bar in Figure S39A) – the coupling of **S2** and **S3** to form **S4** – is shown in Figure S39B. RFR successfully placed **SL2**, which gives 94% yield, in the second suggestion. While LRRF failed to include **SL2** in its prediction, it included **SL4** which gave 87% yield. It is similar to **SL2**, also being an electron-poor aniline-derived 2-oxoacetic acid.

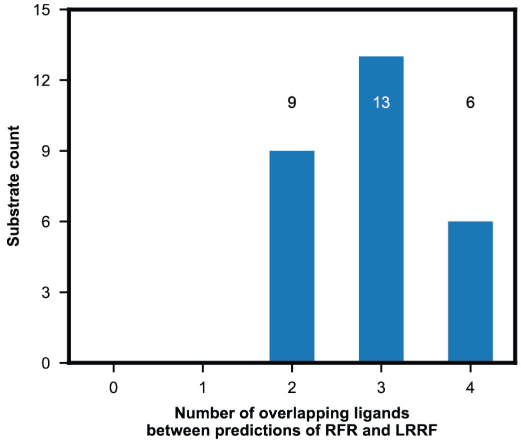

Figure S 40. Number of overlapping ligands between the sets of four ligands suggested by RFR and LRRF.

One observation similar to the example where LRRF's benefit was largest (Figure 6D) was three out of four ligands (**SL1**, **SL3** and **SL4**) overlap between RFR's and LRRF's list of suggestions. Accordingly, the number of overlapping ligands across the two models' predictions were analyzed (Figure S40). There were at least two ligands overlapping between the list of ligands suggested by the two models. In nearly three quarters of all cases (19 out of 28), the predictions were either completely the same or differed by a single ligand. Although this shows the subtle difference between RFR's and LRRF's predictions, the difference in MRR (Figure 6A) and resulting yields (Figure S39A) support the consideration of LRRF over RFR for reactions when more possible conditions are present.

3 References

1. Nielsen, M. K., Ahneman, D. T., Riera, O. & Doyle, A. G. Deoxyfluorination with Sulfonyl Fluorides: Navigating Reaction Space with Machine Learning. *J. Am. Chem. Soc.* 2018, **140,** 5004–5008.

2. Gesmundo, N. J., Sauvagnat, B., Curran, P. J., Richards, M. P., Andrews, C. L., Dandliker, P. J. & Cernak, T. Nanoscale Synthesis and Affinity Ranking. *Nature,* 2018, **557,** 228–232.

3. Landrum, G. A. RDKit. *RDKit: Open-Source Cheminformatics* at <http://www.rdkit.org>

4. Epifanovsky, E., Gilbert, A. T. B., Feng, X., Lee, J., Mao, Y., Mardirossian, N., Pokhilko, P., White, A. F., Coons, M. P., Dempwolff, A. L., Gan, Z., Hait, D., Horn, P. R., Jacobson, L. D., Kaliman, I., Kussmann, J., Lange, A. W., Lao, K. U., Levine, D. S., Liu, J., McKenzie, S. C., Morrison, A. F., Nanda, K. D., Plasser, F., Rehn, D. R., Vidal, M. L., You, Z.-Q., Zhu, Y., Alam, B., Albrecht, B. J., Aldossary, A., Alguire, E., Andersen, J. H., Athavale, V., Barton, D., Begam, K., Behn, A., Bellonzi, N., Bernard, Y. A., Berquist, E. J., Burton, H. G. A., Carreras, A., Carter-Fenk, K., Chakraborty, R., Chien, A. D., Closser, K. D., Cofer-Shabica, V., Dasgupta, S., Wergifosse, M. de, Deng, J., Diedenhofen, M., Do, H., Ehlert, S., Fang, P.-T., Fatehi, S., Feng, Q., Friedhoff, T., Gayvert, J., Ge, Q., Gidofalvi, G., Goldey, M., Gomes, J., González-Espinoza, C. E., Gulania, S., Gunina, A. O., Hanson-Heine, M. W. D., Harbach, P. H. P., Hauser, A., Herbst, M. F., Vera, M. H., Hodecker, M., Holden, Z. C., Houck, S., Huang, X., Hui, K., Huynh, B. C., Ivanov, M., Jász, Á., Ji, H., Jiang, H., Kaduk, B., Kähler, S., Khistyaev, K., Kim, J., Kis, G., Klunzinger, P., Koczor-Benda, Z., Koh, J. H., Kosenkov, D., Koulias, L., Kowalczyk, T., Krauter, C. M., Kue, K., Kunitsa, A., Kus, T., Ladjánszki, I., Landau, A., Lawler, K. V., Lefrancois, D., Lehtola, S., Li, R. R., Li, Y.-P., Liang, J., Liebenthal, M., Lin, H.-H., Lin, Y.-S., Liu, F., Liu, K.-Y., Loipersberger, M., Luenser, A., Manjanath, A., Manohar, P., Mansoor, E., Manzer, S. F., Mao, S.-P., Marenich, A. V., Markovich, T., Mason, S., Maurer, S. A., McLaughlin, P. F., Menger, M. F. S. J., Mewes, J.-M., Mewes, S. A., Morgante, P., Mullinax, J. W., Oosterbaan, K. J., Paran, G., Paul, A. C., Paul, S. K., Pavošević, F., Pei, Z., Prager, S., Proynov, E. I., Rák, Á., Ramos-Cordoba, E., Rana, B., Rask, A. E., Rettig, A., Richard, R. M., Rob, F., Rossomme, E., Scheele, T., Scheurer, M., Schneider, M., Sergueev, N., Sharada, S. M., Skomorowski, W., Small, D. W., Stein, C. J., Su, Y.-C., Sundstrom, E. J., Tao, Z., Thirman, J., Tornai, G. J., Tsuchimochi, T., Tubman, N. M., Veccham, S. P., Vydrov, O., Wenzel, J., Witte, J., Yamada, A., Yao, K., Yeganeh, S., Yost, S. R., Zech, A., Zhang, I. Y., Zhang, X., Zhang, Y., Zuev, D., Aspuru-Guzik, A., Bell, A. T., Besley, N. A., Bravaya, K. B., Brooks, B. R., Casanova, D., Chai, J.-D., Coriani, S., Cramer, C. J., Cserey, G., DePrince, A. E., DiStasio, R. A., Dreuw, A., Dunietz, B. D., Furlani, T. R., Goddard, W. A., Hammes-Schiffer, S., Head-Gordon, T., Hehre, W. J., Hsu, C.-P., Jagau, T.-C., Jung, Y., Klamt, A., Kong, J., Lambrecht, D. S., Liang, W., Mayhall, N. J., McCurdy, C. W., Neaton, J. B., Ochsenfeld, C., Parkhill, J. A., Peverati, R., Rassolov, V. A., Shao, Y., Slipchenko, L. V., Stauch, T., Steele, R. P., Subotnik, J. E., Thom, A. J. W., Tkatchenko, A., Truhlar, D. G., Voorhis, T. V., Wesolowski, T. A., Whaley, K. B., Woodcock, H. L., Zimmerman, P. M., Faraji, S., Gill, P. M. W., Head-Gordon, M., Herbert, J. M. & Krylov, A. I. Software for the Frontiers of Quantum Chemistry: An Overview of Developments in the Q-Chem 5 Package. *J. Chem. Phys.* 2021, **155,** 084801.

5. Jorner, K. *MORFEUS*. at <https://github.com/digital-chemistry-laboratory/morfeus>

6. Brethomé, A. V., Fletcher, S. P. & Paton, R. S. Conformational Effects on Physical-Organic Descriptors: The Case of Sterimol Steric Parameters. *ACS Catal.* 2019, **9,** 2313–2323.

7. Shim, E., Kammeraad, J. A., Xu, Z., Tewari, A., Cernak, T. & Zimmerman, P. M. Predicting Reaction Conditions from Limited Data Through Active Transfer Learning. *Chem. Sci.* 2022, **13,** 6655–6668.

8. Dreher, S. D. & Krska, S. W. Chemistry Informer Libraries: Conception, Early Experience, and Role in the Future of Cheminformatics. *Acc. Chem. Res.* 2021, **54,** 1586–1596.

9. Koike, T. & Akita, M. Visible-Light Radical Reaction Designed by Ru- and Ir-Based Photoredox Catalysis. *Inorganic Chemistry Frontiers,* 2014, **1,** 562–576.

10. Romero, N. A. & Nicewicz, D. A. Organic Photoredox Catalysis. *Chem. Rev.* 2016, **116,** 10075–10166.

11. Prier, C. K., Rankic, D. A. & MacMillan, D. W. C. Visible Light Photoredox Catalysis with Transition Metal Complexes: Applications in Organic Synthesis. *Chem. Rev.* 2013, **113,** 5322–5363.

12. Samha, M. H., Karas, L. J., Vogt, D. B., Odogwu, E. C., Elward, J., Crawford, J. M., Steves, J. E. & Sigman, M. S. Predicting Success in Cu-Catalyzed C–N Coupling Reactions Using Data Science. *Sci. Adv.* 2024, **10,** eadn3478.

13. Diorazio, L. J., Hose, D. R. J. & Adlington, N. K. Toward a More Holistic Framework for Solvent Selection. *Org. Proc. Res. Dev.* 2016, **20,** 760–773.

14. Stevens, J. M., Li, J., Simmons, E. M., Wisniewski, S. R., DiSomma, S., Fraunhoffer, K. J., Geng, P., Hao, B. & Jackson, E. W. Advancing Base Metal Catalysis through Data Science: Insight and Predictive Models for Ni-Catalyzed Borylation through Supervised Machine Learning. *Organomet.* 2022, **41,** 1847–1864.

15. Pedregosa, F., Varoquaux, G., Gramfort, A., Michel, V., Thirion, B., Grisel, O., Blondel, M., Prettenhofer, P., Weiss, R., Dubourg, V., Vanderplas, J., Passos, A., Cournapeau, D., Brucher, M., Perrot, M. & Duchesnay, É. Scikit-learn: Machine Learning in Python. *J. Mach. Learn. Res.* 2011, **12,** 2825–2830.

16. Hüllermeier, E., Fürnkranz, J., Cheng, W. & Brinker, K. Label Ranking by Learning Pairwise Preferences. *Artif. Intell.* 2008, **172,** 1897–1916.

17. Abdi, H. in *Encyclopedia of Measurement and Statistics* (ed. Salkind, N.) 508–510 (SAGE, 2007).

18. Skubi, K. L., Blum, T. R. & Yoon, T. P. Dual Catalysis Strategies in Photochemical Synthesis. *Chem. Rev.* 2016, **116,** 10035–10074.

19. Zhu, C., Yue, H., Chu, L. & Rueping, M. Recent Advances in Photoredox and Nickel Dual-Catalyzed Cascade Reactions: Pushing the Boundaries of Complexity. *Chem. Sci.* 2020, **11,** 4051–4064.

20. Nippa, D. F., Atz, K., Hohler, R., Müller, A. T., Marx, A., Bartelmus, C., Wuitschik, G., Marzuoli, I., Jost, V., Wolfard, J., Binder, M., Stepan, A. F., Konrad, D. B., Grether, U., Martin, R. E. & Schneider, G. Enabling Late-Stage Drug Diversification by High-Throughput Experimentation with Geometric Deep Learning. *Nat. Chem.* 2023, 1–10.
